# ﻿Three new species of *Macrostomum* (Platyhelminthes, Macrostomorpha) from China and Australia, with notes on taxonomy and phylogenetics

**DOI:** 10.3897/zookeys.1099.72964

**Published:** 2022-05-03

**Authors:** Yongshi Shi, Zhiyu Zeng, Jia Wang, Siyu Zhang, Li Deng, Antai Wang

**Affiliations:** 1 Shenzhen Key Laboratory of Marine Bioresource and Eco-environmental Science, College of Life Sciences and Oceanography, Shenzhen University, Shenzhen 518055, China Shenzhen University Shenzhen China

**Keywords:** COI, flatworm, taxonomy, 18S rDNA, 28S rDNA

## Abstract

In this paper, three species of the macrostomid free-living flatworm genus *Macrostomum* are described. Two species, *Macrostomumlittorale* Wang & Shi, **sp. nov.** and *M.shekouense* Wang & Shi, **sp. nov.**, were collected from coastal water at Shenzhen, Guangdong Province, China. One species, *M.brandi* Wang & Shi, **sp. nov.**, was collected from Perth, Western Australia and Queenscliff, Victoria, Australia. *Macrostomumlittorale***sp. nov.** differs from congeneric species within the genus in the length of the stylet, diameter of stylet opening, and the bend of the stylet. *Macrostomumshekouense***sp. nov.** and *M.brandi***sp. nov.** differ from similar species within the genus in the stylet morphology, position of the female antrum and developing eggs, or presence or absence of the false seminal vesicle. Phylogenetic analysis based on cytochrome c oxidase subunit I (COI) gene shows that *M.littorale***sp. nov.** and *M.hystrix* are sister clades on two well-separated branch, *M.shekouense***sp. nov.** and *M.brandi***sp. nov.** are sister clades on two well-separated branches. Accordingly, both morphological and molecular evidence support *M.littorale***sp. nov.**, *M.shekouense***sp. nov.**, and *M.brandi***sp. nov.** as three new species.

## ﻿Introduction

*Macrostomum* Schmidt, 1848 is a genus of the family Macrostomidae (Platyhelminthes; Macrostomorpha), with more than 160 species described to date from around the world (Tyler et al. 2006–2021; Zhang et al. 2021). These free-living flatworms are transparent, hermaphroditic, mostly 1–2 mm in body length, and have a relatively simple general anatomy. *Macrostomum* is attracting interest because it contains *Macrostomumlignano* Ladurner, Schärer, Salvenmoser & Rieger, 2005, a versatile model organism increasingly used in evolutionary, developmental, and molecular biology (Ladurner et al. 2005; Ladurner et al. 2008; Mouton et al. 2009; Vizoso et al. 2010; Wasik et al. 2015; Wudarski et al. 2020). A recent study revealed that the unusual karyotype of *M.lignano* hinders it from becoming a full-fledged genomic model organism, and *M.cliftonense* Schärer & Brand, 2020 was suggested as the potential primary *Macrostomum* model to replace *M.lignano* (Schärer et al. 2020). To explore and describe new species of *Macrostomum* will obviously be of benefit to these research fields by providing more biological and genetic resources since *Macrostomum* is a species-rich genus (Tyler et al. 2006–2021; Brand et al. 2022a).

Taxonomy of the genus *Macrostomum* is particularly challenging, due to the difficulty of their study. In addition, there is considerable convergent evolution of the copulatory organ morphology, particularly in the morphology of penis stylet (Schärer et al. 2011; Brand et al. 2022b). The penis stylet for *Macrostomum* species is considered as the most typical and significant taxonomic feature for identification (Ferguson 1954; Rieger 1977; Rieger et al. 1994). According to morphology of the genital organs and mating behavior, species can be divided into two groups showing a hypodermic mating syndrome (HMS) or reciprocal mating syndrome (RMS) (Schärer et al. 2011). HMS species have extremely similar stylet morphologies, showing a clear case of convergent evolution, which means that those species are hard to distinguish based on stylet morphology only (Schärer et al. 2011). Some molecular markers, such as nuclear ribosomal RNA genes (18S rDNA and 28S rDNA), were used to determine the molecular phylogenetic placement or help identify *Macrostomum* species. However, the rDNA regions are too conserved to successfully resolve interrelationships between some *Macrostomum* species, such as *M.lignano* and *M.janickei* Schärer, 2020 (Schärer et al. 2020). We therefore also used a partial (mitochondrial) cytochrome c oxidase I (COI) gene sequence, a more rapidly evolving marker that was suggested to be used as potential molecular marker resolving interrelationships between species in the Macrostomorpha by several authors (Janssen et al. 2015; Xin et al. 2019; Schärer et al. 2020; Zhang et al. 2021). However, the sequence of COI gene is available for approximately only ten *Macrostomum* species. To understand the evolutionary relationships within *Macrostomum*, much greater species representation would be required.

The first species of *Macrostomum* described from China was *Macrostomumintermedium* Tu, 1934 (Tu 1934). Seventy years later, our laboratory reported a second *Macrostomum* species, *Macrostomumxiamense* Wang & Luo, 2004. Since then, 22 species of *Macrostomum* have been reported from China, of which 21 species were newly described, 13 from freshwater and eight from brackish environments (Lin et al. 2017a; Xin et al. 2019; Zhang et al. 2021). China is likely to have a high richness and diversity of *Macrostomum* species, since 14 new species were reported in the last five years from south China alone, mainly in Guangdong Province.

In this paper, we describe three new species of *Macrostomum*, two species from China and one from Australia. Twenty COI gene sequences of seven species are provided, and phylogenetic analyses inferred from partial 18S rDNA, 28S rDNA, and COI gene sequences of *Macrostomum* taxa are presented.

## ﻿Materials and methods

### ﻿Sample collection and rearing

Specimens of *Macrostomumlittorale* sp. nov. and *M.shekouense* sp. nov. were collected in 2018 from Waterlands Resort located at the estuary of Pearl River in the west of Shenzhen (22°43.32'N, 113°45.88'E) and from the seashore at Shekou peninsula (22°28.77'N, 113°55.12'E), Guangdong Province, China, respectively.

Samples were collected by washing off sediment and organisms from floating plants or underwater stones and using 750-, 125-, and 75-μm mesh nets sequentially. The material retained by the 125-μm and 75-μm mesh nets was transported to the laboratory. All living flatworms were maintained in the water of the original location with a 12:12 h light/dark period at room temperature (25 ± 1 °C). The flatworms were fed with *Paramecium* sp. every two days.

In addition to our own specimens, we also analyzed specimens of *M.brandi* sp. nov. that were previously deposited by Brand et al. (2022a) under the name *Macrostomum* sp. 81 (also called *M.* sp. 81 or Mac081), since it was found to be a close relative of *M.shekouense* sp. nov. These specimens of *M.brandi* sp. nov. were collected in 2017 from Perth, Western Australia (31°59.22'S, 115°49.72'E) (Suppl. material 4: Fig. S1C) and Queenscliff, Victoria (38°16.20'S, 144°38.34'E) (Suppl. material 4: Fig. S1D), Australia (see below for more details).

### ﻿Specimen preparation, observations, and data processing

The procedures of specimen preparation followed the method described by Zhang et al. (2021). In brief, the specimens were fixed in Bouin’s solution after being anesthetized successively with 5% (for 1–2 min) and 7% (for 1–2 min) ethanol in habitat water. The dehydrated specimens were embedded in paraffin and serially sectioned along the sagittal plane (thickness 6 μm) using a manual rotary microtome (Leica RM2235, Leica Biosystems, Germany). After being stained with either hematoxylin-eosin (H&E) or modified Cason’s Mallory–Heidenhain stain and hematoxylin (Yang et al. 2020), the specimens were mounted in neutral balsam for histological observation. Lactic acid phenol liquid (lactic acid: phenol = 1:1 in volume) was used for penis stylet dissection.

All the specimens were observed by stereomicroscope (Leica EZ4, Leica Microsystems, Germany) and differential interference microscopy (Olympus BX51, PA, USA). Images were captured and measured with Olympus DP 2-BSW and Image-Pro Plus software v. 6.0. Type material has been deposited in the Institute of Zoology, Chinese Academy of Sciences, Beijing (IZCAS), with the abbreviation PLA–Ma (Platyhelminthes–*Macrostomum*) followed by catalog numbers.

### ﻿DNA extraction, amplification, and sequencing

After being deprived of food for three days, three individuals of each species (non-types) were placed into liquid nitrogen for 15 s for the following DNA extraction. An E.Z.N.A ^TM^ Mollusk DNA Isolation Kit (Omega, Norcross, GA, USA) was used to extract DNA. PCR reactions were performed using KOD One^TM^ PCR Master Mix (TOYOBO Co. LTD, Japan) with a Thermal Cycler (Applied Biosystems 2720, Thermo Fisher Scientific, USA). The primers for 18S rDNA, 28S rDNA and COI sequences and PCR amplification procedures are listed in Table 1. The amplified 18S rDNA, 28S rDNA, and COI fragments were approximately 1250, 1200, and 610 bp long, respectively. PCR products were separated on a 1.2% agarose gel and purified using a Gel DNA Extraction Kit (Chinatopbio, Shenzhen, China), and were inserted into the pESI-Blunt vector (Hieff Clone^TM^ Zero TOPO-Blunt Cloning Kit, Yeasen, Shanghai, China), respectively. The amplified DNA fragments were sequenced by Sanger sequencing by Beijing Genomics Institute (BGI, Shenzhen, China) or Beijing TsingKe Biotech Co., Ltd (Beijing, China).

**Table 1.** Primer sequences and PCR procedures used for amplification and sequencing.

Gene	Primers	Direction	Sequence(5’-3’)	PCR procedures	References
18S rDNA	Macro_18S_200F	forward	GGCGCATTTATTAGATCAAAACCA	94 °C (2 min); 40× [94 °C (30 s), 54 °C (30 s), 72 °C (2 min)] ; 72 °C (7 min)	Schärer et al. (2011)
	Macro_18S_1640R	reverse	GCAAGCCCCGATCCCTGTC		
28S rDNA	ZX-1	forward	ACCCGCTGAATTTAAGCATAT	95 °C (5 min); 40× [95 °C (30 s), 55 °C (30 s), 72 °C (2 min)] ; 72 °C (7 min)	
	1500R	reverse	GCTATCCTGAGGGAAACTTCG		
COI	Mac_COIF	forward	GTTCTACAAATCATAAGGATATTGG	94 °C (1 min); 5× [94 °C (30 s) , 45 °C (90 s), 72 °C (60 s)] ; 35× [94 °C (30 s) , 51 °C (90 s), 72 °C (60 s)] ; 72 °C (5 min)	Janssen et al. (2015)
	Mac_COIR	reverse	TAAACYTCWGGGTGACCAAAAAACCA		
	F-MS-COI	forward	GGATATTGGWACHTTDTATTT	98 °C (3 min); 20× [98 °C (15 s), 52–42 °C (5 s), 72 °C (10 s)]; 30× [98 °C (15 s), 42 °C (5 s), 72 °C (10 s)]; 72 °C (5 min)	this study
	R-MS-COI	reverse	TTHCGATCWGTTAAHAACAT		

### ﻿Molecular phylogenetic analyses

Newly obtained sequences have been deposited in the GenBank database at NCBI. Sequences used for phylogenetic analyses in this study were obtained from GenBank under accession numbers shown in Suppl. material 1, 2: Tables S1, S2. In total, 43 18S rDNA sequences, 54 28S rDNA sequences, and 46 COI sequences from 42 *Macrostomum* species were included. The 18S rDNA and 28S rDNA of five species of *Psammomacrostomum* and one COI sequence of *Psammomacrostomum* sp. 5 were selected as outgroups for rDNA and COI trees, respectively.

Alignments were performed with the online version of the software MAFFT v. 7 (Kuraku et al. 2013; Katoh et al. 2017), applying the E-INS-i interactive refinement method. Surprisingly, a single-base deletion was found in the COI sequences of *Macrostomumlittorale* sp. nov., *M.hystrix* Örsted, 1843, *M.* sp. 34, *M.taurinum* Wang & Zhang, 2021, and *M.zhujiangense* Wang & Fang, 2016 when it was translated by ORFfinder in NCBI with the genetic code 9, and we discuss this observation in more detail below. For alignment and analysis, the missing bases were filled with N. Ambiguous sites (e.g., containing gaps and poorly aligned sites) were removed by Gblocks v. 0.91b (Castresana 2000) with default settings. The final length of the aligned 18S rDNA, 28S rDNA, and COI sequences were 1195, 776, and 406 bp, respectively. Uncorrected pairwise distances between species were calculated in MEGA v. 6.06 (Tamura et al. 2013). Uncorrected genetic distances (GDs) for COI were calculated based on all sequences obtained from *M.littorale* sp. nov., *M.shekouense* sp. nov., and *M.brandi* sp. nov. together with those used for phylogenetic tree calculation (alignment length: 406 bp). A concatenated dataset (18S–28S rDNA) was combined using MEGA v. 6.06 (Tamura et al. 2013); missing sequences are denoted as Ns in the concatenated alignment. A substitution saturation test was carried out in DAMBE v. 5 (Xia and Lemey 2009; Xia 2017) to assess the nucleotide substitution saturation.

Based on the Akaike information criterion (AIC), we used ModelFinder (Kalyaanamoorthy et al. 2017) to find the best evolution model for maximum likelihood (ML) method; the GTR+F+I+G4 model was selected for all datasets. Meanwhile, to find the best-fit model for Bayesian inference (BI) analyses, MrModelTest v. 2.3 (Nylander 2004) was used applying the AIC; GTR+I+G model was chosen for all datasets. The gene partitions in the concatenated dataset were defined as 18S rDNA/28S rDNA. PartitionFinder v. 2.1.1 (Lanfear et al. 2017) was used to select the best-fit models for each partition based on AIC; for ML and BI methods, the models for both 18S rDNA and 28S rDNA were GTR+I+G. After that, phylogenetic trees were constructed by both ML and BI. For ML, analyses were performed in IQ-TREE v. 1.6.2 (Nguyen et al. 2015) with 1000 bootstrap replicates. Model parameters were calculated independently for each gene partition of the concatenated dataset. For BI, analyses were performed in MrBayes v. 3.2.6 (Ronquist et al. 2012) with two simultaneous runs of one cold and three heated chains. Partitioned analysis was performed on the concatenated dataset. The Markov Chain Monte Carlo (MCMC) algorithm was run for 1,500,000 generations for all datasets in four simultaneous chains. Every 1000^th^ generation was sampled. Burn-in was chosen as 25% of the results. Effective sample size (ESS) values of each parameter in the .*p* files were checked by TRACER v. 1.7.1 (Rambaut et al. 2018) to ensure good convergence. All trees were visualized using FigTree v. 1.4.3 (Rambaut 2009) and Adobe Photoshop CC 2017.

## ﻿Results

### ﻿Molecular phylogeny

**Figure 1.**
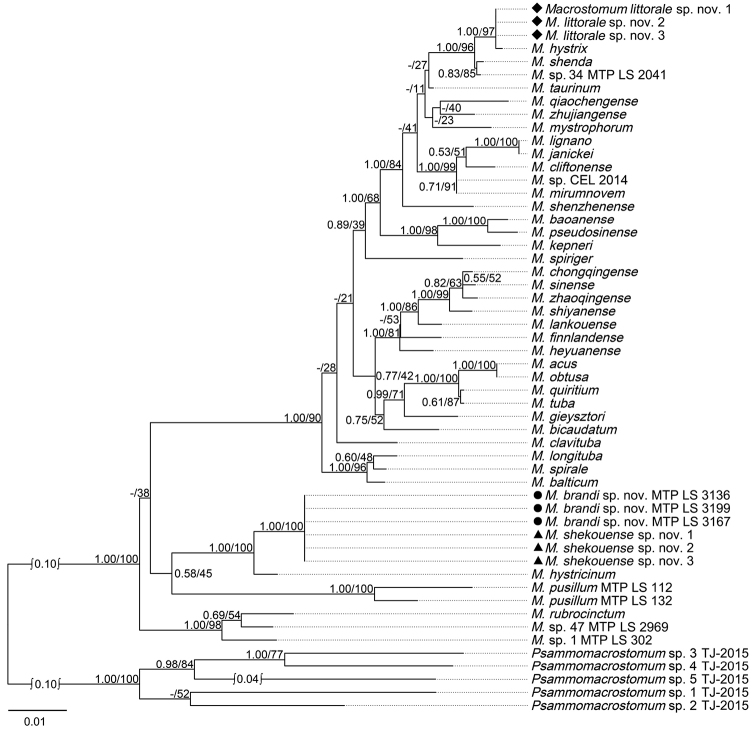
Maximum likelihood phylogenetic tree topology based on the 18S–28S rDNA dataset. Numbers on branches indicate support values (posterior probability/bootstrap). Scale bar: substitutions per nucleotide position.

**Figure 2.**
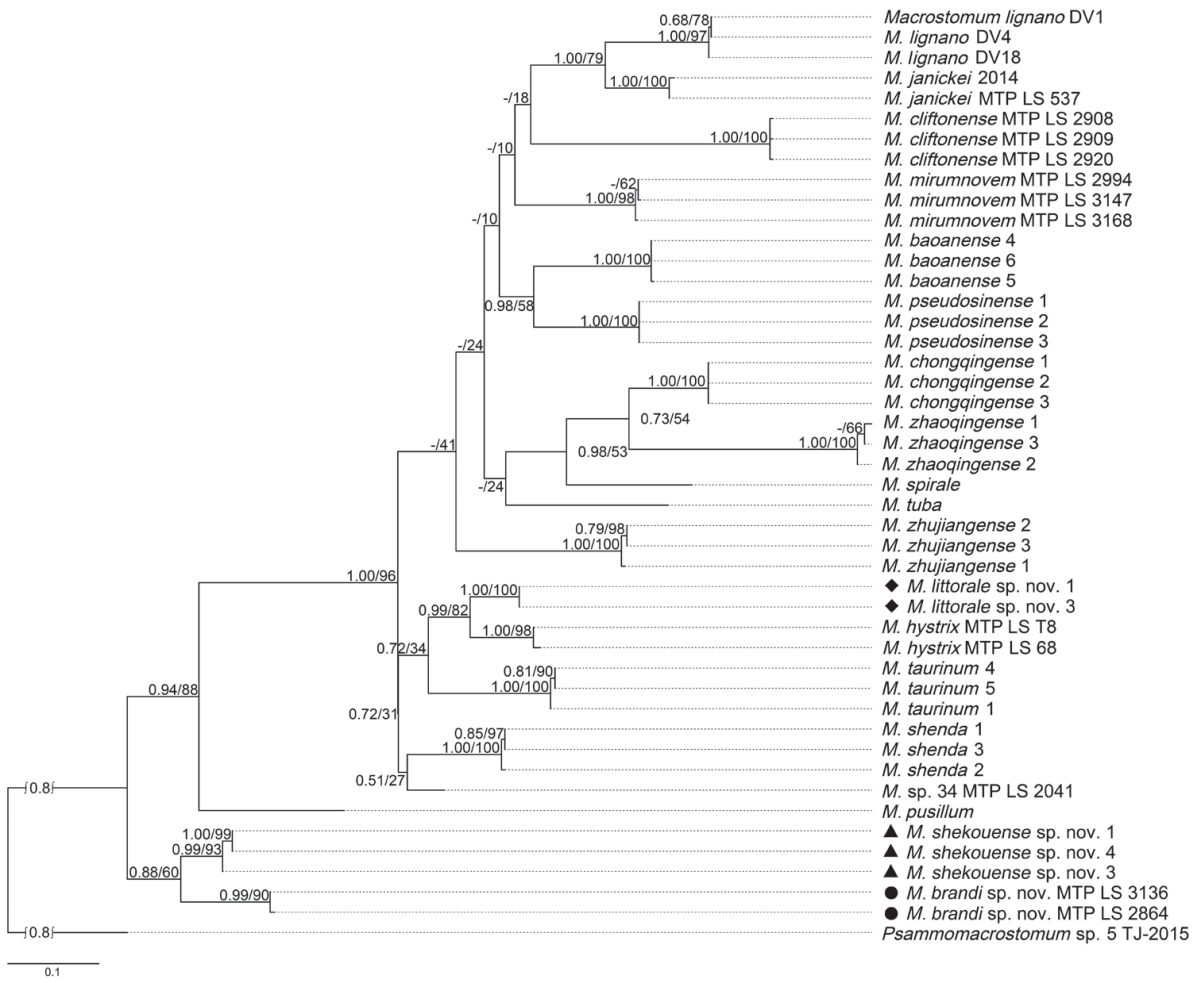
Maximum likelihood phylogenetic tree topology based on the COI dataset. Numbers on branches indicate support values (posterior probability/bootstrap). Scale bar: substitutions per nucleotide position. Note: the COI sequences of *M.brandi* sp. nov. MTP LS 3136 (SAMN15061113), *M.brandi* sp. nov. MTP LS 2864 (SAMN15061091) and *M.* sp. 34 MTP LS 2041 (SAMN15061043) are extracted from deposited transcriptomes (Brand et al. 2022a).

Twenty fragments of sequences of COI were amplified and sequenced from two specimens of *Macrostomumlittorale* sp. nov., and 18 specimens of the other six *Macrostomum* species, *M.baoanense* Wang & Fang, 2016, *M.pseudosinense* Wang & Zhang, 2021, *M.shenda* Wang & Xin, 2019, *M.shekouense* sp. nov., *M.taurinum*, *M.zhujiangense*, using three specimens of each species. Three fragments of sequences of COI were obtained from deposited transcriptome assemblies obtained from individual specimens of *M.brandi* sp. nov. (MTP LS 3136, SAMN15061113; MTP LS 2864, SAMN15061091) and *M.* sp. 34 (MTP LS 2041, SAMN15061043), respectively (Brand et al. 2022a). The results of phylogenetic analyses are shown in Figs 1, 2, and Suppl. material 5, 6: Figs S2, S3. The values of the posterior probability from the BI analyses were added to ML consensus trees, since the topology of phylogenetic trees from the BI analyses and the ML analyses are congruent, both when inferred from rDNA sequences or from COI sequences (Figs 1, 2, Suppl. material 5, 6: Figs S2, S3).

The individuals of *Macrostomumlittorale* sp. nov. and *M.hystrix* fell into one supported clade with high support values (1.00 PP, 97% BP) in the phylogenetic tree resulting from the analysis of the concatenated 18S and 28S rDNA dataset (Fig. 1). The individuals of *M.shekouense* sp. nov. cluster together with *M.brandi* sp. nov. with high support values (1.00 PP, 100% BP) in the phylogenetic tree resulting from the analysis of the concatenated dataset (Fig. 1). However, in the more rapidly evolving COI gene tree, *M.littorale* sp. nov. occupied a separate branch by 0.99 PP, 82% BP, supporting a separation between *M.littorale* sp. nov. and its congener, *M.hystrix* (Fig. 2). Similarly, the individuals of *M.shekouense* sp. nov. clustered in a clade with high support values (0.99 PP, 93% BP), as a sister clade of *M.brandi* sp. nov. (Fig. 2).

### ﻿Systematic account

**Macrostomorpha Doe, 1986**

**Family Macrostomidae Beneden E, 1870**

#### Genus *Macrostomum* Schmidt, 1848

##### ﻿ Macrostomum littorale

Taxon classificationAnimaliaMacrostomidaMacrostomidae

Wang & Shi sp. nov.

05B28189-176A-5EF9-8332-327420FC0108

http://zoobank.org/B06653BF-4D32-4C53-AB47-659F0CBC6655

Figs 3 , 4 , 5

###### Type material.

Two specimens: holotype IZCASPLA–Ma0140, collected by Fan Xin from Waterlands Resort located at the estuary of pearl River in the west of Shenzhen, Guangdong, China (22°43.32'N, 113°45.88'E) in April 2018, unsectioned whole-body mounted in neutral balsam. The paratype (collection date and locality same as the holotype), is one serially-sectioned specimen mounted in neutral balsam (IZCASPLA–Ma0141). Digital photomicrographs of the holotype specimen and the sectioned paratype specimen, as well as photomicrographs of five non-type specimens (IZCASPLA–Ma0140a-e) imaged in vivo, were deposited on the Macrostomorpha Taxonomy and Phylogeny website (at https://macrostomorpha.myspecies.info) and can also be accessed at https://doi.org10.5281/zenodo.4585492.

###### Habitat.

Specimens were collected from Waterlands Resort at the estuary of the Pearl River at Shenzhen, Guangdong Province, China (Suppl. material 4: Fig. S1A). The animals were collected from floating plants. Salinities of the water in habitat were 9‰–11‰.

###### Diagnosis.

**Figure 3.**
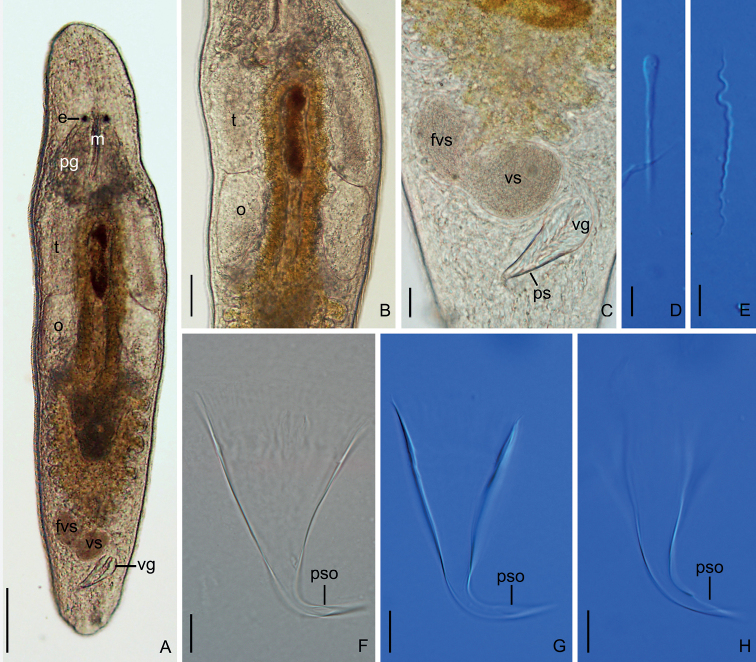
*Macrostomumlittorale* Wang & Shi, sp. nov. **A** whole animal, ventral view **B** testes and ovaries **C** male copulatory apparatus, ventral view **D** immature sperm **E** mature sperm **F–H** penis stylet. Abbreviations: e: eye; fvs: false vesicula seminalis; m: mouth; o: ovary; pg: pharyngeal glands; ps: penis stylet; pso: penis stylet opening; t: testis; vg: vesicula granulorum; vs: vesicula seminalis. Scale bars: 100 μm (**A**); 50 μm (**B**); 20 μm (**C**); 5 μm (**D, E**); 10 μm (**F–H**).

A *Macrostomum* species with dorsoventrally flattened body, two almost round eyes, and rounded rostrum (Fig. 3A). Body length and width of mature worms are 920 ± 109 μm and 192 ± 49 μm, respectively. Testes clearly larger than ovaries. Stylet (62 ± 7.9 μm) is a hook-like and gradually narrowing funnel with an 105° bend in the 66% position (when measured from the proximal to the distal part). The stylet opening is 6 ± 0.9 μm in diameter, willow leaf-shaped, located on the concave side of the subterminal region of the stylet (Fig. 3F, G, H). Muscular walls of vesicula seminalis and vesicula granulorum thickened. No bristles and brush on sperm (35 ± 1.1 μm) (Fig. 3E).

###### Etymology.

The name of this new species is derived from its habitat.

###### Description.

Body dorsoventrally flattened, colorless. Mature individual 920 ± 109 μm in length and 192 ± 49 μm in width (*n* = 6) (Fig. 3A). Entire body covered with cilia (6 ± 1.3 μm in length, *n* = 6). Tufts of sensory hairs, 7 ± 1.1 μm (*n* = 6) long, sparsely distributed along body edges. Anterior and posterior edges of body equipped with rigid cilia, 6 ± 1.0 μm (*n* = 6) long. The rhabdite rods scattered in groups (mostly 5–7 rhabdites in each group) on the body surface, most abundant on the dorsal side. Two round eyes, separated from each other by a distance of 37 ± 6.1 μm (*n* = 6) (Fig. 3A). Pharynx surrounded by abundant gland cells on both sides, mouth 73 ± 16 μm (*n* = 6) in length (Fig. 3A).

**Figure 4.**
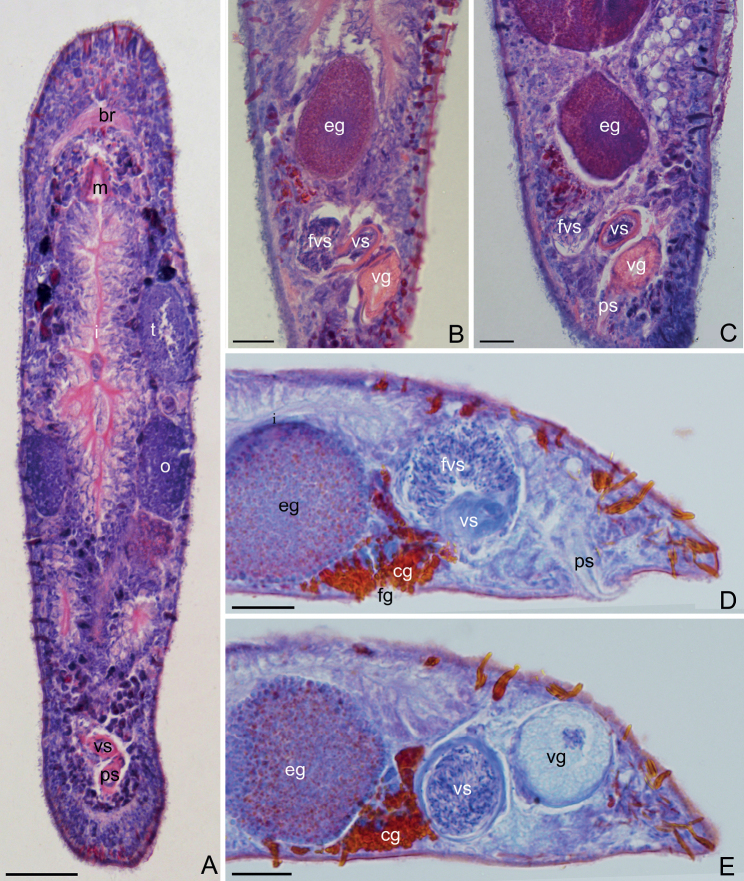
*Macrostomumlittorale* Wang & Shi, sp. nov. **A–C** horizontal whole-body sections, ventral view (stained by H&E) **D, E** longitudinal sections, showing male copulatory apparatus (stained by modified Mallory-Heidenhain/Cason stain and hematoxylin). Abbreviations: br: brain; cg: cement glands; eg: egg; fg: female gonopore; fvs: false vesicula seminalis; i: intestine; m: mouth; o: ovary; ps: penis stylet; t: testis; vg: vesicula granulorum; vs: vesicula seminalis. Scale bars: 50 μm (**A**); 20 μm (**B–E**).

**Figure 5.**
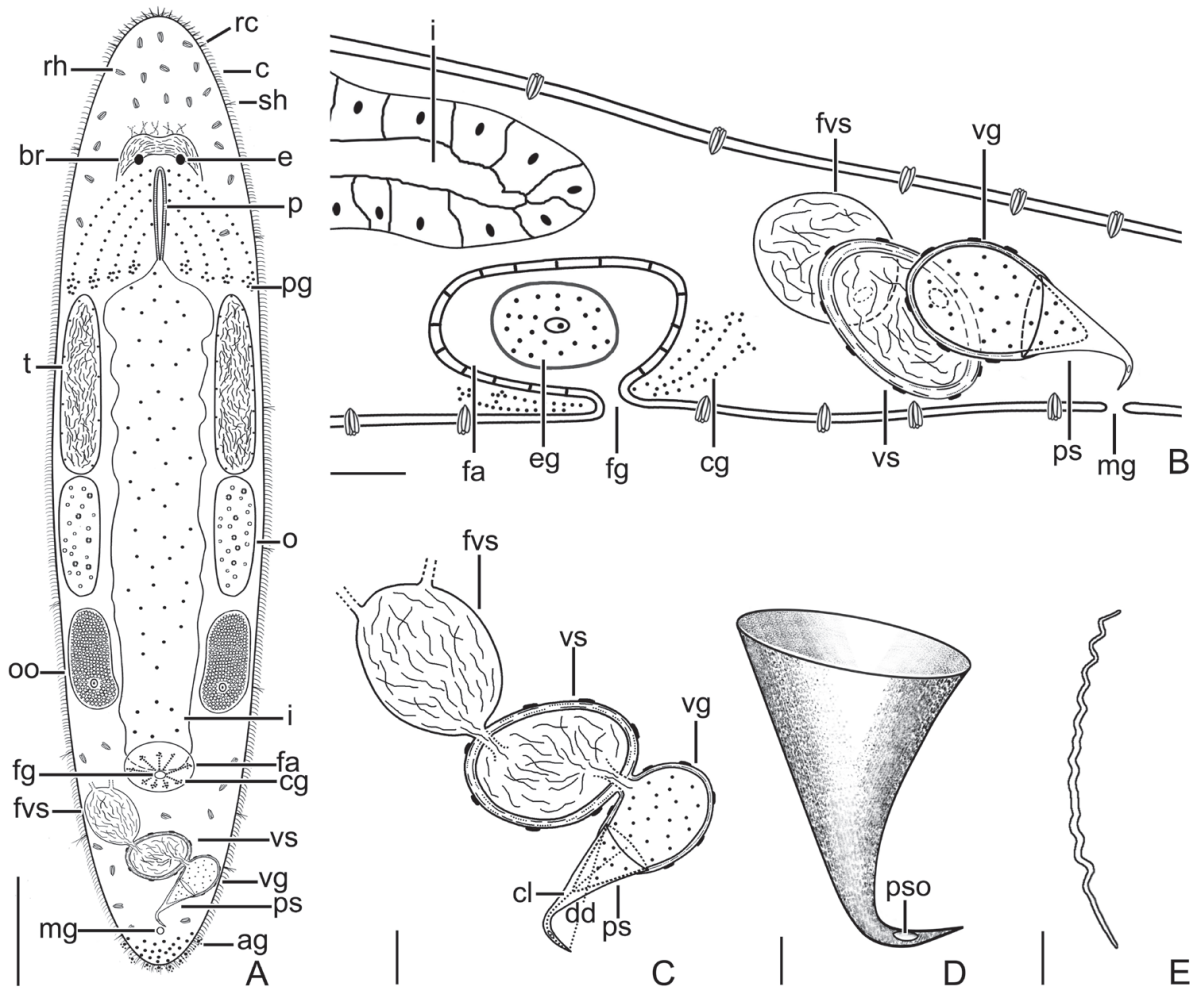
*Macrostomumlittorale* Wang & Shi, sp. nov. **A** whole body, ventral view **B** sagittal section of the tail **C** male copulatory apparatus **D** penis stylet **E** mature sperm. Abbreviations: ag: adhesive glands; br: brain; c: cilia; cl: curved length from proximal to distal ends; cg: cement glands; dd: direct distance between proximal and distal ends; e: eye; eg: egg; fa: female antrum; fg: female gonopore; fvs: false vesicula seminalis; i: intestine; mg: male gonopore; o: ovary; oo: oocyte; p: pharynx; pg: pharyngeal glands; ps: penis stylet; pso: penis stylet opening; rc: rigid cilia; rh: rhabdites; sh: sensory hair; t: testis; vg: vesicula granulorum; vs: vesicula seminalis. Scale bars: 100 μm (**A**); 20 μm (**B, C**); 10 μm (**D**); 5 μm (**E**).

Paired elliptic testes, 154 ± 7.9 μm (*n* = 5) in length and 40 ± 13 μm (*n* = 5) in width (Figs 3B, 4A, 5A). Male copulatory apparatus consisting of false vesicula seminalis, vesicula seminalis, vesicula granulorum and stylet (Figs 3C, 4B–E, 5A–C). False vesicula seminalis oval-shaped, located behind female antrum, connecting to vesicula seminalis at its left side from ventral view. Vesicula granulorum connecting to oval-shaped vesicula seminalis on the right rear part from ventral view, while extending into proximal opening of penis stylet on the other side. Both vesicula seminalis and vesicula granulorum have a thickened muscular wall (Figs 3C, 4B–E, 5A–C). Stylet 35 ± 6.3 μm (*n* = 5) in diameter at its proximal opening; curved length from proximal to distal ends (dotted line ‘cl’ in Fig. 5C) 64 ± 7.4 μm (*n* = 5); direct distance between proximal and distal ends (dotted line ‘dd’ in Fig. 5C) 62 ± 7.9 μm (*n* = 5). Stylet hook-like, gradually narrowing from the proximal end, curved at 66% position from proximal end with bending angle of 105° (*n* = 5) (Figs 3F–H, 5D); The stylet opening 6 ± 0.9 μm (*n* = 5) in diameter, willow leaf-shaped, located at the concave side of the subterminal region of stylet (Figs 3F–H, 5D). Mature sperm 35 ± 1.1 μm (*n* = 5) in length, having neither bristle nor brush. The boundary between feeler, sperm body and sperm shaft not clear (Figs 3E, 5E).

Pair of oval ovaries, 115 ± 9.9 μm (*n* = 6) in length and 44 ± 8.9 μm (*n* = 6) in width, located on both sides of intestine (Figs 3B, 5A). Female gonopore opening ventrally at female antrum, surrounded by numerous cement glands.

###### Remarks.

A comparison between *Macrostomumlittorale* sp. nov. in this study and eleven similar species (stylet hook-like) within the genus is shown in Table 2.

**Table 2.** Comparison between the new species and the similar species with hook-like stylets within the genus.

Species	Body Length (μm)	Female antrum position	Stylet length ^a^ (μm)	Diameter of stylet opening (proximal / distal μm)	Penis stylet opening (pso) position *	Bending angle (°) and position of curve in stylet*	Habitat	Distribution	Reference
*M.astericis*	800	posterior	25–32	13–16/2.7*	65%/convex	93°/50%	Marine	Galapagos, Ecuador	Schmidt and Sopott-Ehlers (1976)
*M.hystricinum*	NA	posterior	32	22/4–6	81%/convex	85°/81%	Brackish	Widely distributed	Beklemischev (1951) and Wang et al. (2017)
M.hystrix*	NA	posterior	44	20/5	78%/convex	85°/75%	Brackish	Italy	Schärer et al. (2011) and Brand et al (2022a)
*M.littorale* sp. nov.	920 ± 109	posterior	64 ± 7.4	35 ± 6.3/6 ± 0.9	85%/concave	105°/66%	Brackish	China	this study
*M.shekouense* sp. nov.	978 ± 143	50% of body length	46 ± 3.5	22 ± 2.7/3 ± 0.3	73%/convex	90°/65%	Brackish	China	this study
*M.brandi* sp. nov.	1147 ± 151	50% of body length	55 ± 5.0	37 ± 9/2.4 ± 0.05	70%/convex	90°/70%	Marine	Australia	*M.* sp. 81 in Brand et al (2022a)
*M.obelic*is	1,000–2,000	50% of body length	35–50	16–25/NA	77%/convex	90°/69%	Marine	Galapagos, Ecuador	Schmidt and Sopott-Ehlers (1976)
*M.peteraxi*	1,500	posterior	27–30	12.5/NA	NA	90°/NA	Marine	Romania	Mack-Fira (1971)
*M.pusillum*	500–800	posterior	24–26	9–12/NA	NA	90°/42%	Marine	Germany	Ax (1951)
*M.qiaochengense*	1,147 ± 52	posterior	51 ± 3.5	21 ± 1.2/20 ± 1.6	63%/convex	90°/63%	Brackish	China	Wang et al. (2017)
*M.rubrocinctum*	1,500–2,000	posterior	55	30/NA	NA	90°/67%	Marine	Germany	Ax (1951)
*M.* sp 1 MTP LS 302*	NA	posterior	42	23/4.4	73%/convex	105°/60%	NA	Italy	Schärer et al. (2011)

*Measurement based on images and scales given in the references. The procedures of measuring the angle refer to Ferguson (1940). NA: Not available, information cannot be obtained from the literature. a: Stylet length refers to curved length (cl) from proximal to distal ends.

The main difference between *M.littorale* sp. nov. and *M.astericis* Schmidt & Sopott-Ehlers, 1976, *M.qiaochengense* Wang & Fang, 2017, and *M.* sp. 1 is the location of penis stylet opening (pso), which is at the concave side of the curved tube in *M.littorale* sp. nov., while it is located at the convex side of the curved tube in the other species. Moreover, the length of the stylet in *M.hystricinum* and *M.astericis* is shorter than that in *M.littorale* sp. nov. The distal opening of *M.qiaochengense* (diameter 20 ± 1.6 μm) is much larger than that of *M.littorale* sp. nov. (diameter 6 ± 0.9 μm).

The length of the stylet in *M.peteraxi* Mack-Fira, 1971 (27–30 μm) and *M.pusillum* (24–26 μm) is smaller than that in *M.littorale* sp. nov (64 ± 7.4 μm). Furthermore, the stylet proximal opening in *M.peteraxi* (diameter 12.5 μm) and *M.pusillum* (diameter 9–12 μm) is much smaller than that in *M.littorale* sp. nov. (diameter 35 ± 6.3 μm).

The bending angle of the stylet in *M.rubrocinctum* Ax, 1951 (90°) is smaller than that in *M.littorale* sp. nov. (105°). The body length of *M.rubrocinctum* (1,500–2,000 μm) is much larger than that in *M.littorale* sp. nov. (920 ± 109 μm). Moreover, *M.rubrocinctum* has a red pigmented ring on its head, which is absent in *M.littorale* sp. nov.

Accordingly, it is evident that *M.littorale* sp. nov. is a new species within the genus *Macrostomum* based on morphology in combination with phylogenetic analyses.

##### ﻿ Macrostomum shekouense

Taxon classificationAnimaliaMacrostomidaMacrostomidae

Wang & Shi sp. nov.

1EDE451D-7005-55BB-A419-99A17C9CD0D0

http://zoobank.org/190A7664-768C-4D1F-BBE8-A1CA316D26D4

Figs 6 , 7 , 8

###### Type material.

Three specimens: holotype (stained by H&E) IZCASPLA–Ma0150, collected by Linhong Zhong in October 2018 from the seashore at Shekou peninsula, Guangdong, China (22°28.77'N, 113°55.12'E), the unsectioned whole body mounted in neutral balsam. The paratypes (collection date and locality same as holotype), comprising two serially-sectioned specimens mounted in neutral balsam (IZCASPLA–Ma0151–152). Digital photomicrographs of the holotype specimen and the sectioned paratype specimens, as well as photomicrographs of four non-type specimens (IZCASPLA–Ma0150a-d) imaged in vivo, were further deposited on the Macrostomorpha Taxonomy and Phylogeny website (at https://macrostomorpha.myspecies.info) and can also be accessed at https://doi.org10.5281/zenodo.4585492.

###### Habitat.

Specimens were collected from the seashore at Shekou peninsula (Suppl. material 4: Fig. S1B). The animals were collected from underwater stones. Salinities of the water in habitat were 16‰–21‰.

###### Diagnosis.

**Figure 6.**
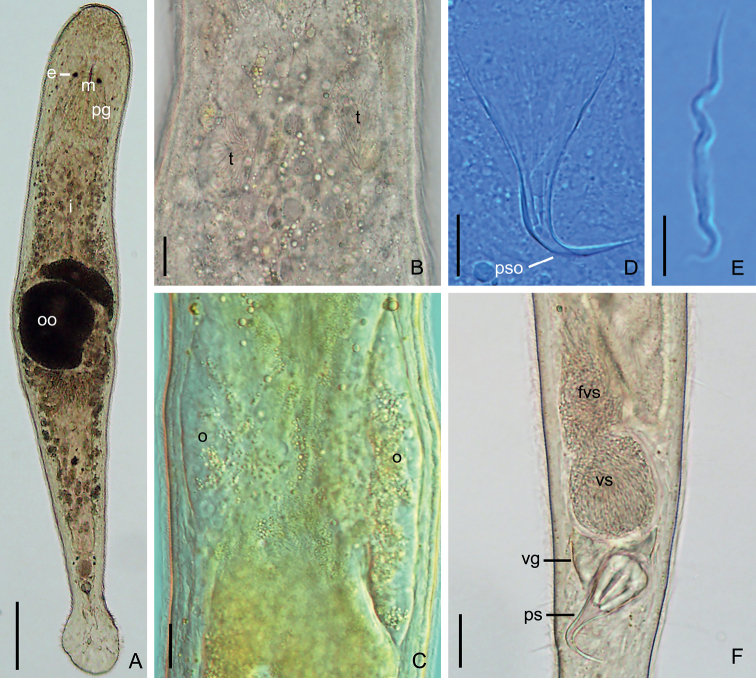
*Macrostomumshekouense* Wang & Shi, sp. nov. **A** whole animal, ventral view **B** testes **C** ovaries **D** penis stylet **E** mature sperm **F** male copulatory apparatus, ventral view. Abbreviations: e: eye; fvs: false vesicula seminalis; i: intestine; m: mouth; o: ovary; oo: oocyte; pg: pharyngeal glands; ps: penis stylet; pso: penis stylet opening; t: testis; vg: vesicula granulorum; vs: vesicula seminalis. Scale bars: 100 μm (**A**); 20 μm (**B, C, F**); 5 μm (**D, E**).

*Macrostomum* with slightly dorsoventrally flattened body and wider arc-shaped tail (Fig. 6A). Muscular wall of both vesicula seminalis and vesicula granulorum thickened. The stylet, hook-like in shape, is a gradually narrowing funnel, including a 90° bending at the 65% position from proximal end (Fig. 6D). No bristles and brush on sperm (Fig. 6E).

###### Etymology.

The specific epithet refers to the locality where the species was found, which is the Shekou peninsula, Guangdong, China.

###### Description.

**Figure 7.**
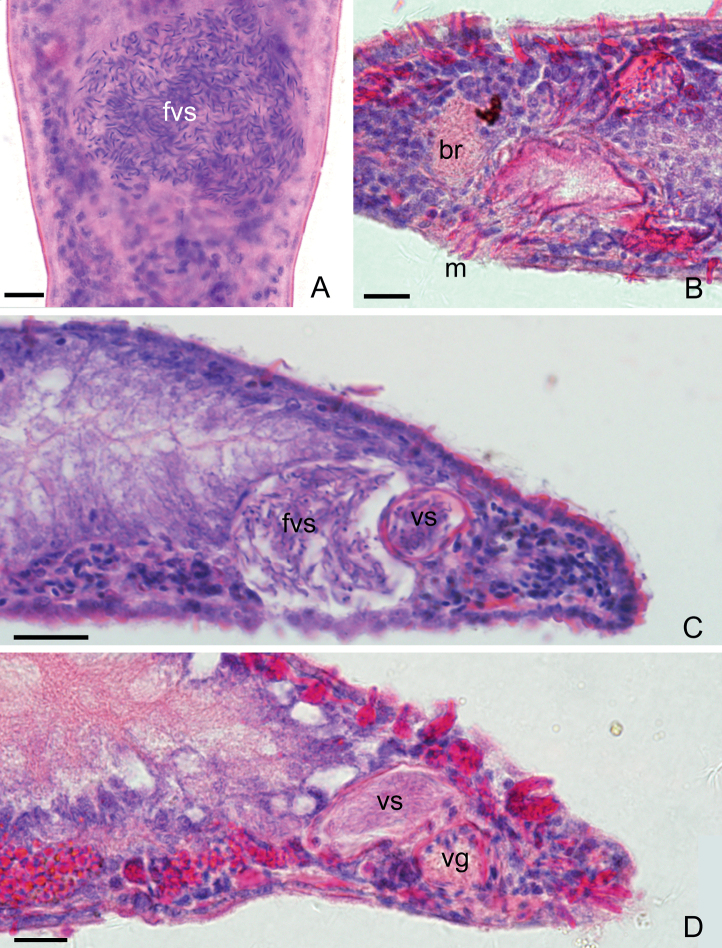
*Macrostomumshekouense* Wang & Shi, sp. nov. **A** mounted specimen, ventral view **B–D** longitudinal whole-body sections **A–D** stained by H&E. Abbreviations: br: brain; fvs: false vesicula seminalis; m: mouth; vg: vesicula granulorum; vs: vesicula seminalis. Scale bars: 10 μm (**A–C**); 20 μm (**D**).

Mature worms 978 ± 143 μm in length and 115 ± 18 μm in width, covered with dense cilia 4 ± 0.7 μm in length (*n* = 7) (Fig. 6A). Two eyes appear cup-shaped in most individuals, kidney-shaped in some individuals. Rigid cilia, 9 ± 0.8 μm (*n* = 7) and 14 ± 1.2 μm (*n* = 5) in length, at anterior and posterior body end, respectively. Sensory hairs, 13 ± 0.8 μm (*n* = 7) in length, sparsely distributed on body edges. Rhabdite rods mainly distributed on the dorsal side of the body. Distance between the two eyes 23 ± 1.5 μm (*n* = 5) (Fig. 6A). Mouth 83 ± 3.8 μm (*n* = 5) in length (Figs 6A, 7B).

**Figure 8.**
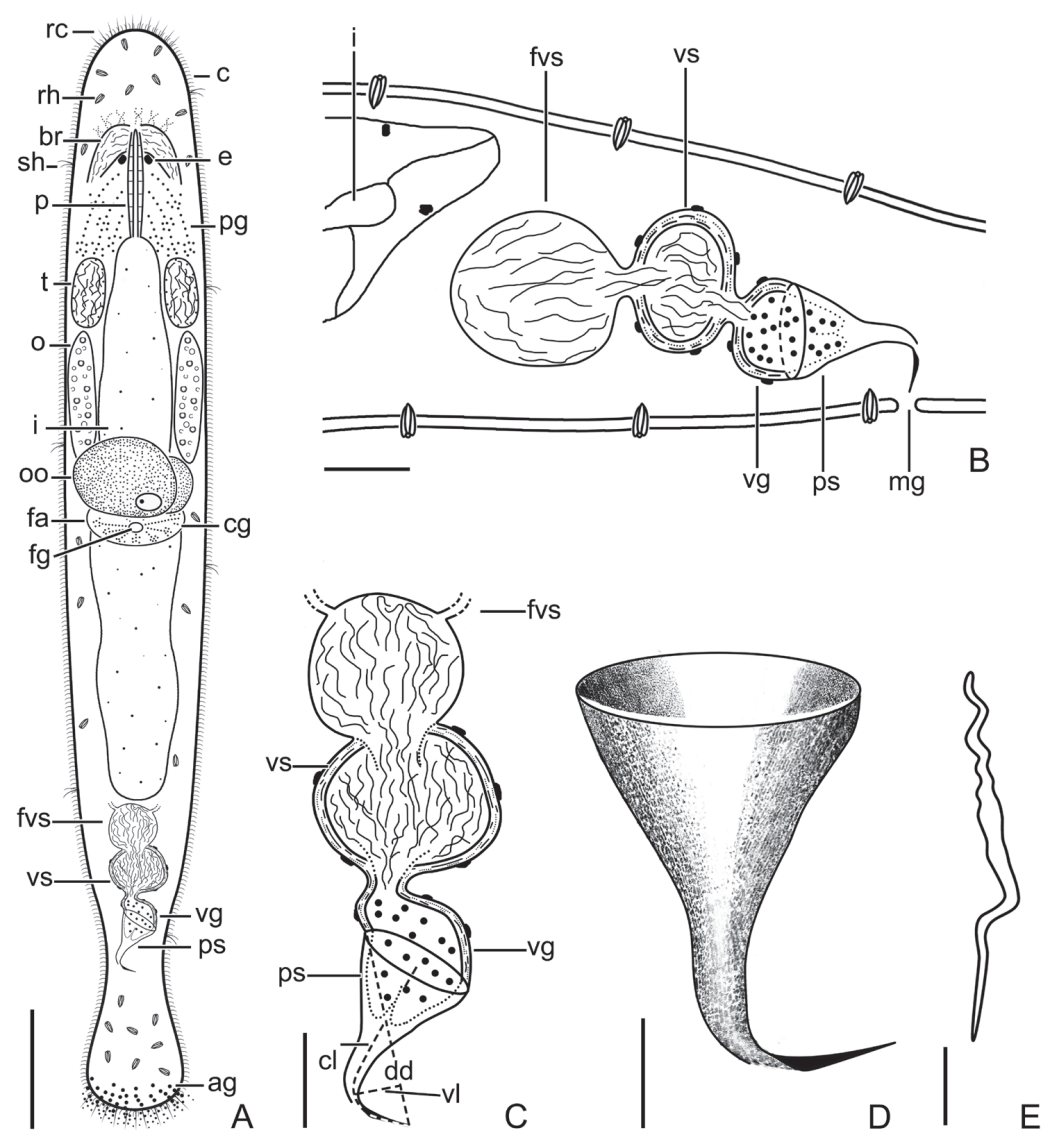
*Macrostomumshekouense* Wang & Shi, sp. nov.: **A** whole body, ventral view **B** sagittal section of the tail **C** male copulatory apparatus **D** penis stylet **E** mature sperm. Abbreviations: ag: adhesive glands; br: brain; c: cilia; cl: curved length from proximal to distal ends; cg: cement glands; dd: direct distance between proximal and distal ends; e: eye; fa: female antrum; fg: female gonopore; fvs: false vesicula seminalis; i: intestine; mg: male gonopore; o: ovary; oo: oocyte; p: pharynx; pg: pharyngeal glands; ps: penis stylet; rc: rigid cilia; rh: rhabdites; sh: sensory hair; t: testis; vl: vertical line from line “dd” to curve vertex of line “cl”; vg: vesicula granulorum; vs: vesicula seminalis. Scale bars: 100 μm (**A**); 20 μm (**B, C**); 10 μm (**D**); 5 μm (**E**).

Testes oval inshape, 70 ± 9.0 μm (*n* = 7) in length and 23 ± 5.2 μm (*n* = 7) in width, located on the ventral side of the intestine and situated closely behind the pharynx (Figs 6B, 7A). Male copulatory apparatus consisting of false vesicula seminalis, vesicula seminalis, vesicula granulorum, and penis stylet. False vesicula seminalis oval-shaped, located at the posterior of the body. Vesicula seminalis oval-shaped, connecting to false vesicula seminalis on one side, while connecting to vesicula granulorum on the other side. Muscular wall of both vesicula seminalis and vesicula granulorum thickened. Vesicula granulorum extended into proximal end of penis stylet (Figs 6F, 7C, D, 8B, C). The stylet, hook-like in shape, is a gradually narrowing funnel, including a 90° bending in the 65% position (Fig. 6D); proximal opening 22 ± 2.7 μm (*n* = 6) in diameter; curved length from proximal to distal ends (dotted line ‘cl’ in Fig. 8C) 46 ± 3.5 μm (*n* = 6); direct distance between the proximal and distal ends (dotted line ‘dd’ in Fig. 8C) 39 ± 2.9 μm (*n* = 6); vertical line from line “dd” to the curve vertex of line “cl” (dotted line ‘vl’ in Fig. 8C) 12.7 ± 0.94 μm (*n* = 5). Stylet has an opening (diameter 3 ± 0.3 μm (*n* = 4) located at the convex side of the subterminal region of stylet and a pointed distal thickening (Figs 6D, 8D). Sperm, 27 ± 0.8 μm in length, having neither bristles nor brush. The boundary between feeler, sperm body, and sperm shaft is not clear (Figs 6E, 8E).

Pair of short elliptic ovaries, 101 ± 16 μm (*n* = 7) in length and 19 ± 4.7 μm (*n* = 7) in width, located on both sides of intestine (Figs 6B, 8A). Female antrum located at the ventral side at 50% of body length. Female gonopore surrounded by numerous cement glands.

##### ﻿ Macrostomum brandi

Taxon classificationAnimaliaMacrostomidaMacrostomidae

Wang & Shi sp. nov.

FD62FCF1-07B7-5B86-B169-CDACDA6295A7

http://zoobank.org/53CB59EA-8C1F-4B5D-B9CC-2F2D1FBC1535

Figs 9 , 10

###### Type material.

*Macrostomumbrandi* sp. nov. was first collected in 2017 by Brand et al. (2022a) and therein referred to as *Macrostomum* sp. 81 (as well as *M.* sp. 81 and Mac081). The present description of the species is based on photomicrographs and videos that Brand et al. (2022a) deposited with multiple specimens of the species. As the holotype we designate their transcriptome-sequenced specimen MTP LS 2864 (transcriptome accession SAMN15061091). The digital type materials of the specimen are available on Zenodo (https://zenodo.org/record/5656981) (for details see Brand et al. 2022a).

###### Habitat.

The type specimen was collected from shallow subtidal sediment in Perth (Suppl. material 4: Fig. S1C). The longitude and latitude of the sampling site was described in part *Sample collection and rearing*. Salinities of the water in the habitat was 30‰.

###### Diagnosis.

**Figure 9.**
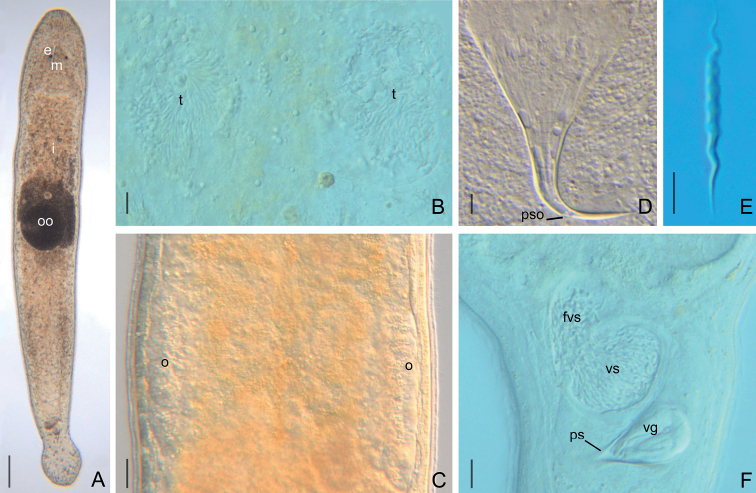
*Macrostomumbrandi* Wang & Shi, sp. nov. **A** whole animal, ventral view **B** testes **C** ovaries **D** penis stylet **E** mature sperm **F** male copulatory apparatus, ventral view. Abbreviations: e: eye; fvs: false vesicula seminalis; m: mouth; o: ovary; oo: oocyte; ps: penis stylet; pso: penis stylet opening; t: testis; vg: vesicula granulorum; vs: vesicula seminalis. Scale bars: 100 μm (**A**); 10 μm (**B, D**); 20 μm (**C, F**); 5 μm (**E**).

**Figure 10.**
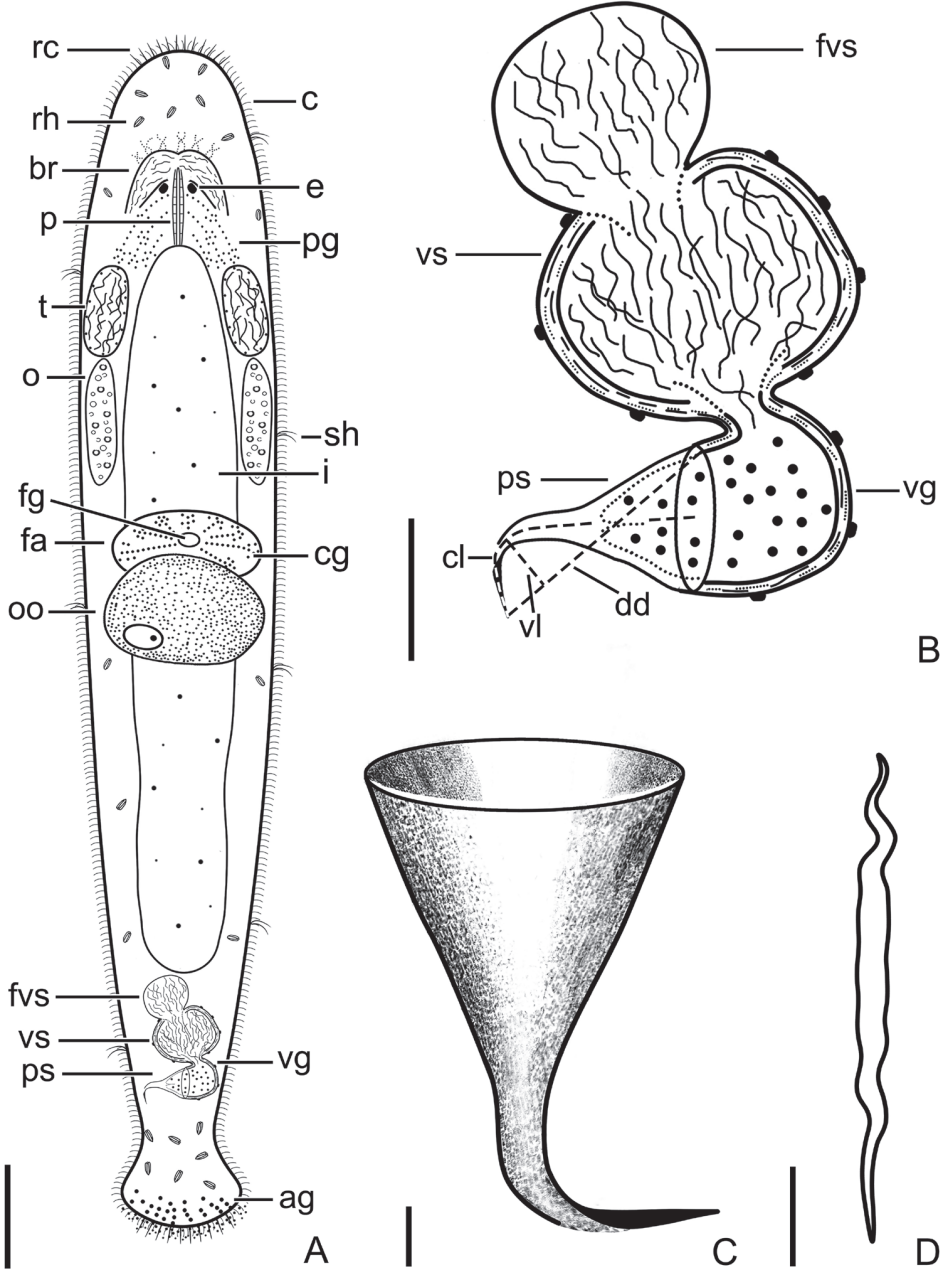
*Macrostomumbrandi* Wang & Shi, sp. nov. **A** whole body, ventral view **B** sagittal section of the tail **C** male copulatory apparatus **D** penis stylet **E** mature sperm. Abbreviations: ag: adhesive glands; br: brain; c: cilia; cl: curved length from proximal to distal ends; cg: cement glands; dd: direct distance between proximal and distal ends; e: eye; fa: female antrum; fg: female gonopore; fvs: false vesicula seminalis; i: intestine; mg: male gonopore; o: ovary; oo: oocyte; p: pharynx; pg: pharyngeal glands; ps: penis stylet; rc: rigid cilia; rh: rhabdites; sh: sensory hair; t: testis; vl: vertical line from line “dd” to curve vertex of line “cl”; vg: vesicula granulorum; vs: vesicula seminalis. Scale bars: 100 μm (**A**); 20 μm (**B**); 5 μm (**C, D**).

A *Macrostomum* species with slightly dorsoventrally flattened body and wider arc-shaped tail (Figs 9A, 10A). Both vesicula seminalis and vesicula granulorum have a thickened muscular wall. The stylet, hook-like in shape, is a gradually narrowing funnel, including a 90° bending at the 70% position (Figs 9D, 10C). No bristles and brush on sperm (Figs 9E, 10D).

###### Etymology.

Species name in honor of Lukas Schärer’s former PhD student Jeremias Brand, with whom Lukas Schärer collected this species in both Perth, Western Australia and Queenscliff, Victoria, Australia.

###### Description.

Mature individuals 1147 ± 151 μm in length and 229 ± 48 μm in width. Two eyes appear kidney-shaped in most individuals, circular in some individuals. Distance between two eyes 37 ± 10 μm (*n* = 15) (Fig. 9A). Mouth 55 ± 18 μm (*n* = 9) in length (Fig. 9A). The body is covered homogeneously with cilia, 8 ± 1.7 μm in length (*n* = 12). Rigid cilia are 12 ± 2.1 μm (*n* = 15) and 9 ± 2.0 μm (*n* = 16) in length at the anterior and posterior body end, respectively. Tufts of sensory hairs sparsely distributed along the body edges. Rhabdite rods mainly distributed on the dorsal side of the body (Fig. 9A).

Paired elliptic testes, 89 ± 22 μm (*n* = 5) in length and 53 ± 30 μm (*n* = 5) in width, located on the ventral side of the intestine and situated closely behind the pharynx (Figs 9B, 10A). Male copulatory apparatus consisting of false vesicula seminalis, vesicula seminalis, vesicula granulorum, and penis stylet. False vesicula seminalis oval-shaped, located at the posterior of the body. It connects on the rear part to an oval-shaped muscular vesicula seminalis. The vesicula seminalis is connected anterolaterally to the muscular vesicula granulorum. Muscular wall of both vesicula seminalis and vesicula granulorum thickened. Vesicula granulorum extended into proximal end of penis stylet (Figs 9F, 10B). The stylet is a hook-like and gradually-narrowing funnel with a 90° bending at the 70% position (Figs 9D, 10C); proximal opening 37 ± 9 μm (*n* = 7) in diameter; curved length from proximal to distal ends (dotted line ‘cl’ in Fig. 10B) 55 ± 5.0 μm (*n* = 7); direct distance between proximal and distal ends (dotted line ‘dd’ in Fig. 10B) 49 ± 5 μm (*n* = 7); vertical line from line “dd” to curve vertex of line “cl” (dotted line ‘vl’ in Fig. 10B) 9 ± 1 μm (*n* = 7). The stylet opening 2.5 ± 0.05 μm (*n* = 6) in diameter, located at the convex side of the subterminal region of stylet and it has a pointed distal thickening (Figs 9D, 10C). Mature sperm without bristles and brush, 24 ± 2 μm in length. The boundary between feeler, sperm body, and sperm shaft is not clear (Figs 9E, 10D).

Pair of ovaries lie directly behind the testes and show the short elliptic shape, 124 ± 31 μm (*n* = 8) in length and 30 ± 7 μm (*n* = 8) in width (Figs 9C, 10A). Female gonopore opening ventrally at female antrum, surrounded by numerous cement glands. Female antrum lies at the ventral side at 50% of body length.

###### Remarks.

A comparison between *Macrostomumshekouense* sp. nov., *M.brandi* sp. nov., and ten similar species with hook-like stylets within the genus is shown in Table 2. For the 12 listed species, the female antrum of only three species (*M.shekouense* sp. nov., *M.brandi* sp. nov., and *M.obelicis*) lies at the ventral side at 50% of body length, while that of the other nine species lies considerably further towards the posterior.

Based on the 28S rDNA phylogenetic tree in Brand et al. (2022a) it seems clear that there currently are no other known species that are very close to *Macrostomumbrandi* sp. nov. *M.shekouense* sp. nov., *M.brandi* sp. nov., and *M.obelicis* are very similar in stylet morphology, particularly with respect to the position of the stylet opening, as well as the bending angle and position of the curve in the stylet, although the stylet is a little larger in *M.brandi* sp. nov. than that in *M.shekouense* sp. nov. and in *M.obelicis*. In terms of overall morphology of the flatworms, *M.shekouense* sp. nov. is much more similar to *M.brandi* sp. nov. This includes a central position of the developing eggs at ~ 50% (rather than an even more anterior position at ~ 40% in *M.obelicis*), and the linear anterior-posterior arrangement of the false seminal vesicle, the true seminal vesicle, and a posteriorly pointing stylet (while the *M.obelicis* lacks a false seminal vesicle, and has a seminal vesicle and stylet that are oriented laterally in an opposite direction). In agreement with this, the molecular phylogenetic analyses based on 18S–28S rDNA show that *M.shekouense* sp. nov. is a very close relative of *M.brandi* sp. nov.

However, in the COI gene tree, *M.shekouense* sp. nov. occupied a separate branch by 0.99 PP, 93% BP, supporting a separation between *M.shekouense* sp. nov. and *M.brandi* sp. nov. (Fig. 2). GDs based on the COI sequence within the genus *Macrostomum* were also calculated, showing that GDs between individuals of *M.shekouense* sp. nov. and *M.brandi* sp. nov. were between 10.1% and 10.9% (Suppl. material 3: Table S3), while the two specimens from Perth and Queenscliff differed by only 0.2%. Hebert et al. (2003) found that GDs between species are ordinarily greater than 3% for a range of invertebrates. Moreover, we note that *M.littorale* sp. nov. and *M.hystrix* show a clearly different stylet morphology, while the GD between these two species (8.9%) is less than that between *M.shekouense* sp. nov. and *M.brandi* sp. nov.

In addition, we calculated and compared the ratio of the length of two lines of the stylet as shown in Fig. 8 and Fig. 10, dd and vl. This suggests that the dd-to-vl ratio in *M.brandi* sp. nov. (5.2 ± 0.62, *n* = 7) is significantly higher than that in *M.shekouense* sp. nov. (3.1 ± 0.26, *n* = 5) (2-tail p < 0.001, Independent Sample Test) (Suppl. material 7: Fig. S4).

Accordingly, both morphological and molecular evidence supports that *M.shekouense* sp. nov. and *M.brandi* sp. nov. are two new species.

## ﻿Discussion

It is a challenging task to identify *Macrostomum* species for various reasons. Firstly, these small, fragile microturbellarians are difficult to study. Secondly, there is considerable convergent evolution of the copulatory organ morphology in *Macrostomum* species, particularly the morphology of the penis stylet (e.g., Schärer et al. 2011; Brand et al. 2022b). Brand et al. (2022a) suggested that investigations of species within the hypodermic clade without support from molecular data thus require considerable caution due to the striking cases of convergent evolution found in HMS species. Molecular markers such as the mitochondrial COI, showing a more rapid evolutionary rate than nuclear 18S and 28S rDNA, was suggested to better resolve species-level relationships (Schärer et al. 2020). However, mitochondrial COI are currently not available in most species of *Macrostomum* to date. The present study has now identified a primer pair, which was used to amplify and sequence eight new mitochondrial COI sequences of three *Macrostomum* species, as well as extracting three COI sequences from previously deposited transcriptomes (Suppl. material 2: Table S2). The COI gene tree supported a separation between *M.janickei* and *M.lignano* (see also Schärer et al. 2020), and it supported separation between *M.littorale* sp. nov. and *M.hystrix*, as well as between *M.shekouense* sp. nov. and *M.brandi* sp. nov., although in all these cases these species pairs could not be separated by 18S and 28S rDNA trees (Figs 1, 2). Accordingly, with an increase in the number of COI sequences provided for *Macrostomum* species, molecular phylogenetic analyses based on mitochondrial COI gene could be a useful tool for helping to identify *Macrostomum* species in combination with morphological characters. In addition, it is suggested that mitochondrial COI sequences of *Macrostomum* species could be amplified using primer pair provided either by Janssen et al. (2015) or by this study showing in Table 1.

In this study, a significant difference was found in dd-to-vl ratio of stylet in *M.shekouense* sp. nov. and *M.brandi* sp. nov. We propose that the dd-to-vl ratio of stylet could serve as an additional character for the delimitation of the hypodermic species with hook-like stylets, considering that the values of dd and vl vary with the change of stylet length, the bending angle and position of the curve in the stylet, and the diameter of proximal opening of stylet.

**Figure 11.**
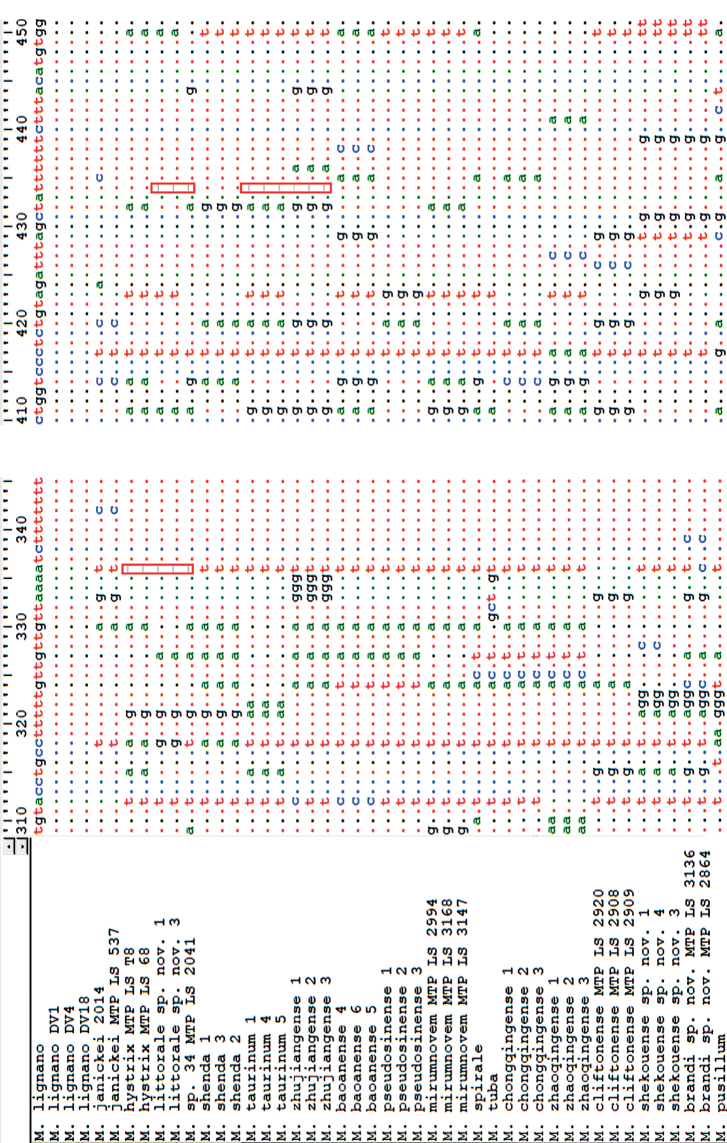
Alignment of mitochondrial COI sequences showing the position of nucleotide base deletion.

It is interesting to note that the COI gene sequences of both *M.littorale* sp. nov. and *M.* sp. 34 show single-base deletions at two positions, position 336 and position 434 when counting from the ATT start codon of the 1,548 bp COI gene of *M.lignano* (Egger et al. 2017, GenBank: MF078637), leading to a frameshift mutation and a TAG stop codon only ten bases (and three more stop codons in the sequenced fragment alone). Furthermore, two species, *M.taurinum* and *M.zhujiangense*, show a single-base deletion at position 434, resulting in the frameshift mutation of ten amino acid residues followed by a stop codon (Fig. 11). A similar phenomenon was previously reported in *M.hystrix* (Schärer et al. 2020), for which the COI gene sequence shows a single-base deletion at position 336, leading to a frameshift mutation and a TAG stop codon only ten bases on (and eight more stop codons in the sequenced fragment alone). The single-base deletion at position 336 or at position 434 would be expected to lead to a truncation of the resulting COI protein.

Given the importance of the COI protein in the electron transport chain of mitochondrial oxidative phosphorylation, this finding is surprising and requires further examination and validation, as already noted by Schärer et al. (2020). Further studies are needed to elucidate the phenomenon of single-base deletion in the COI gene of *Macrostomum* species. It will be interesting to further explore the mechanism of gene expression of the COI gene in the *Macrostomum* species, particularly the post-transcriptional processing of precursor-messenger RNA, since at least in *M.hystrix* the frames-shifted transcript seems to be abundantly expressed (Schärer et al. 2020).

## Supplementary Material

XML Treatment for Macrostomum littorale

XML Treatment for Macrostomum shekouense

XML Treatment for Macrostomum brandi

## Supplementary materials

Supplementary material 1 Table S1 Data type docx file Explanation note GenBank accession numbers of 18S and 28S rDNA sequences for species taxa used in the phylogenetic analyses. https://binary.pensoft.net/file/679671This dataset is made available under the Open Database License (http://opendatacommons.org/licenses/odbl/1.0/). The Open Database License (ODbL) is a license agreement intended to allow users to freely share, modify, and use this Dataset while maintaining this same freedom for others, provided that the original source and author(s) are credited.Yongshi Shi, Zhiyu Zeng, Jia Wang, Siyu Zhang, Li Deng, Antai Wang

Supplementary material 2 Table S2 Data type docx file Explanation note GenBank accession numbers of COI sequences for species taxa used in the phylogenetic analyses. https://binary.pensoft.net/file/679672This dataset is made available under the Open Database License (http://opendatacommons.org/licenses/odbl/1.0/). The Open Database License (ODbL) is a license agreement intended to allow users to freely share, modify, and use this Dataset while maintaining this same freedom for others, provided that the original source and author(s) are credited.Yongshi Shi, Zhiyu Zeng, Jia Wang, Siyu Zhang, Li Deng, Antai Wang

Supplementary material 3 Table S3 Data type xls file Explanation note The genetic distances (GDs) among *Macrostomum* species calculated from COI sequences. https://binary.pensoft.net/file/679673This dataset is made available under the Open Database License (http://opendatacommons.org/licenses/odbl/1.0/). The Open Database License (ODbL) is a license agreement intended to allow users to freely share, modify, and use this Dataset while maintaining this same freedom for others, provided that the original source and author(s) are credited.Yongshi Shi, Zhiyu Zeng, Jia Wang, Siyu Zhang, Li Deng, Antai Wang

Supplementary material 4 Figure S1 Data type jpg file Explanation note Locality and habitat of the different species. https://binary.pensoft.net/file/679674This dataset is made available under the Open Database License (http://opendatacommons.org/licenses/odbl/1.0/). The Open Database License (ODbL) is a license agreement intended to allow users to freely share, modify, and use this Dataset while maintaining this same freedom for others, provided that the original source and author(s) are credited.Yongshi Shi, Zhiyu Zeng, Jia Wang, Siyu Zhang, Li Deng, Antai Wang

Supplementary material 5 Figure S2 Data type jpg file Explanation note Maximum-likelihood phylogenetic tree topology based on 18S rDNA dataset. https://binary.pensoft.net/file/679675This dataset is made available under the Open Database License (http://opendatacommons.org/licenses/odbl/1.0/). The Open Database License (ODbL) is a license agreement intended to allow users to freely share, modify, and use this Dataset while maintaining this same freedom for others, provided that the original source and author(s) are credited.Yongshi Shi, Zhiyu Zeng, Jia Wang, Siyu Zhang, Li Deng, Antai Wang

Supplementary material 6 Figure S3 Data type jpg file Explanation note Maximum-likelihood phylogenetic tree topology based on 28S rDNA dataset. https://binary.pensoft.net/file/679676This dataset is made available under the Open Database License (http://opendatacommons.org/licenses/odbl/1.0/). The Open Database License (ODbL) is a license agreement intended to allow users to freely share, modify, and use this Dataset while maintaining this same freedom for others, provided that the original source and author(s) are credited.Yongshi Shi, Zhiyu Zeng, Jia Wang, Siyu Zhang, Li Deng, Antai Wang

Supplementary material 7 Figure S4 Data type jpg file Explanation note Comparison of value dd/vl between *M.shekouense* sp. nov. and *M.brandi* sp. nov. (2-tail p < 0.001, Independent Sample Test). https://binary.pensoft.net/file/679677This dataset is made available under the Open Database License (http://opendatacommons.org/licenses/odbl/1.0/). The Open Database License (ODbL) is a license agreement intended to allow users to freely share, modify, and use this Dataset while maintaining this same freedom for others, provided that the original source and author(s) are credited.Yongshi Shi, Zhiyu Zeng, Jia Wang, Siyu Zhang, Li Deng, Antai Wang
