# Supplementary material for: ﻿Three new species of Macrostomum (Platyhelminthes, Macrostomorpha) from China and Australia, with notes on taxonomy and phylogenetics
Source: Zookeys. 2022 May 3;1099:1–28. doi: 10.3897/zookeys.1099.72964 (PMC9848920; doi:10.3897/zookeys.1099.72964)
Supplement: Supplementary material 1 — Table S1 [file zookeys-1099-001_article-72964__-s001.docx]

Table S1 GenBank accession numbers of 18S and 28S rDNA sequences for species taxa used in the phylogenetic analyses.

| Species | 18S rDNA | | 28S rDNA | |
| --- | --- | --- | --- | --- |
|  | Accession number | Reference | Accession number | Reference |
| *Macrostomum acus* | KY579352 | Lin et al. (2017a) | KY579343 | Lin et al. (2017a) |
| *M. balticum* | FJ715310 | Schärer et al. (2011) | FJ715330 | Schärer et al. (2011) |
| *M. baoanense* 1 | MT764991 | this study | MT774558 | this study |
| *M. bicaudatum* | — | — | KY652224 | Lin et al. (2017a) |
| *M. brandi* sp. nov. MTP LS 3136 | — | — | MT428806 | Brand et al. (2022a) |
| *M. brandi* sp. nov. MTP LS 3167 | — | — | MT428807 | Brand et al. (2022a) |
| *M. brandi* sp. nov. MTP LS 3199 | — | — | MT428808 | Brand et al. (2022a) |
| *M. chongqingense* | KX769148 | Lin et al. (2017b) | KX769150 | Lin et al. (2017b) |
| *M. clavituba* | FJ715304 | Schärer et al. (2011) | FJ715324 | Schärer et al. (2011) |
| *M. cliftonense* | — | — | MK684171 | Schärer et al. (2020) |
| *M. finnlandense* | FJ715302 | Schärer et al. (2011) | FJ715322 | Schärer et al. (2011) |
| *M. gieysztori* | FJ715301 | Schärer et al. (2011) | FJ715321 | Schärer et al. (2011) |
| *M. heyuanense* | — | — | KY652221 | Lin et al. (2017a) |
| *M. hystricinum* | FJ715311 | Schärer et al. (2011) | FJ715331 | Schärer et al. (2011) |
| *M. hystrix* | FJ715303 | Schärer et al. (2011) | FJ715323 | Schärer et al. (2011) |
| *M. janickei* | — | — | MK684168 | Schärer et al. (2020) |
| *M. kepneri* | FJ715307 | Schärer et al. (2011) | FJ715327 | Schärer et al. (2011) |
| *M. lankouense* | KY814495 | Lin et al. (2017a) | KY814498 | Lin et al. (2017a) |
| *M. lignano* | FJ715306 | Schärer et al. (2011) | FJ715326 | Schärer et al. (2011) |
| *M. littorale* sp. nov. 1 | MT765187 | this study | MT765184 | this study |
| *M. littorale* sp. nov. 2 | MT765188 | this study | MT765185 | this study |
| *M. littorale* sp. nov. 3 | MT765189 | this study | MT765186 | this study |
| *M. longituba* | FJ715309 | Schärer et al. (2011) | FJ715329 | Schärer et al. (2011) |
| *M. mirumnovem* | — | — | MK684172 | Schärer et al. (2020) |
| *M. mystrophorum* | FJ715305 | Schärer et al. (2011) | FJ715325 | Schärer et al. (2011) |
| *M. obtusa* | KY579348 | Xin et al. (2019) | KY579339 | Xin et al. (2019) |
| *M. pseudosinense* | MT758405 | Zhang et al. (2021) | MT758408 | Zhang et al. (2021) |
| *M. pusillum* MTP LS 112 | FJ715313 | Schärer et al. (2011) | FJ715333 | Schärer et al. (2011) |
| *M. pusillum* MTP LS 132 | FJ715314 | Schärer et al. (2011) | FJ715334 | Schärer et al. (2011) |
| *M. qiaochengense* | KY008497 | Wang et al. (2017) | KY008500 | Wang et al. (2017) |
| *M. quiritium* | FJ715299 | Schärer et al. (2011) | FJ715319 | Schärer et al. (2011) |
| *M. rubrocinctum* | KC869789 | Laumer and Giribet (2014) | KC869842 | Laumer and Giribet (2014) |
| *M. shenda* | MG675031 | Xin et al. (2019) | MG675034 | Xin et al. (2019) |
| *M. shenzhenense* | KY008491 | Wang et al. (2017) | KY008494 | Wang et al. (2017) |
| *M. shekouense* sp. nov. 1 | MT765181 | this study | MT765178 | this study |
| *M. shekouense* sp. nov. 2 | MT765182 | this study | MT765179 | this study |
| *M. shekouense* sp. nov. 3 | MT765183 | this study | MT765180 | this study |
| *M. shiyanense* | KY814505 | Lin et al. (2017a) | KY814502 | Lin et al. (2017a) |
| *M. sinense* | KY579354 | Xin et al. (2019) | KY579345 | Lin et al. (2017a) |
| *M.* sp. CEL 2014 | KC869790 | Laumer and Giribet (2014) | KC869843 | Laumer and Giribet (2014) |
| *M.* sp. 1 MTP LS 302 | FJ715312 | Schärer et al. (2011) | FJ715332 | Schärer et al. (2011) |
| *M.* sp. 34 MTP LS 2041 | — | — | MT428635 | Brand et al. (2022a) |
| *M.* sp. 47 MTP LS 2969 | — | — | MT428683 | Brand et al. (2022a) |
| *M. spirale* | FJ715308 | Schärer et al. (2011) | FJ715328 | Schärer et al. (2011) |
| *M. spiriger* | MG675037 | Xin et al. (2019) | MG675040 | Xin et al. (2019) |
| *M. taurinum* | MT758411 | Zhang et al. (2021) | MT758414 | Zhang et al. (2021) |
| *M. tuba* | KP730496 | Janssen et al. (2015) | KP730549 | Janssen et al. (2015) |
| *M. zhaoqingense* | — | — | KX769153 | Lin et al. (2017b) |
| *M. zhujiangense* | KX771197 | Fang et al. (2016) | KY359386 | Wang et al. (2017) |
| *Psammomacrostomum* sp. 1 TJ-2015 | KP730508 | Janssen et al. (2015) | KP730539 | Janssen et al. (2015) |
| *Psammomacrostomum* sp. 2 TJ-2015 | KP730481 | Janssen et al. (2015) | KP730543 | Janssen et al. (2015) |
| *Psammomacrostomum* sp. 3 TJ-2015 | KP730500 | Janssen et al. (2015) | KP730530 | Janssen et al. (2015) |
| *Psammomacrostomum* sp. 4 TJ-2015 | KP730514 | Janssen et al. (2015) | KP730555 | Janssen et al. (2015) |
| *Psammomacrostomum* sp. 5 TJ-2015 | KP730513 | Janssen et al. (2015) | KP730540 | Janssen et al. (2015) |
