# Supplementary material for: ﻿Three new species of Macrostomum (Platyhelminthes, Macrostomorpha) from China and Australia, with notes on taxonomy and phylogenetics
Source: Zookeys. 2022 May 3;1099:1–28. doi: 10.3897/zookeys.1099.72964 (PMC9848920; doi:10.3897/zookeys.1099.72964)
Supplement: Supplementary material 2 — Table S2 [file zookeys-1099-001_article-72964__-s002.docx]

Table S2 GenBank accession numbers of COI sequences for taxa used in the phylogenetic analyses.

| Species | COI | |
| --- | --- | --- |
|  | Accession number | Reference |
| *Macrostomum baoanense* 4 | MW633246 | this study |
| *M. baoanense* 5 | MW633247 | this study |
| *M. baoanense* 6 | MW633248 | this study |
| *M. brandi* sp. nov. MTP LS 2864^c^ | SAMN15061091 | Brand et al. (2022a) |
| *M. brandi* sp. nov. MTP LS 3136^c^ | SAMN15061113 | Brand et al. (2022a) |
| *M. chongqingense* 1 | KY006971 | Lin et al. (2017b) |
| *M. chongqingense* 2 | KY006972 | Lin et al. (2017b) |
| *M. chongqingense* 3 | KY006973 | Lin et al. (2017b) |
| *M. cliftonense* MTP LS 2908 | MK690029 | Schärer et al. (2020) |
| *M. cliftonense* MTP LS 2909 | MK690032 | Schärer et al. (2020) |
| *M. cliftonense* MTP LS 2920 | MK690030 | Schärer et al. (2020) |
| *M. hystrix* MTP LS T8 | MK690020 | Schärer et al. (2020) |
| *M. hystrix* MTP LS 68 | KP730561 | Janssen et al. (2015) |
| *M. janickei* MTP LS 537 | MK690018 | Schärer et al. (2020) |
| *M. janickei* 2014 | MK690038 | Schärer et al. (2020) |
| *M. lignano* DV1 | MK690040 | Schärer et al. (2020) |
| *M. lignano* DV4 | MK690043 | Schärer et al. (2020) |
| *M. lignano* DV18 | MK690047 | Schärer et al. (2020) |
| *M. littorale* sp. nov. 1 | ON207842 | this study |
| *M. littorale* sp. nov. 3 | ON207843 | this study |
| *M. mirumnovem* MTP LS 2994 | MK690019 | Schärer et al. (2020) |
| *M. mirumnovem* MTP LS 3147 | MK690027 | Schärer et al. (2020) |
| *M. mirumnovem* MTP LS 3168 | MK690025 | Schärer et al. (2020) |
| *M. pseudosinense* 1^a^ | MW646906 | this study |
| *M. pseudosinense* 2^a^ | MW646907 | this study |
| *M. pseudosinense* 3^a^ | MW646908 | this study |
| *M. pusillum* | KP730558 | Janssen et al. (2015) |
| *M. shekouense* sp. nov. 1 | MW646918 | this study |
| *M. shekouense* sp. nov. 3 | MW646919 | this study |
| *M. shekouense* sp. nov. 4 | MW646920 | this study |
| *M. shenda* 1^b^ | MW646921 | this study |
| *M. shenda* 2^b^ | MW646922 | this study |
| *M. shenda* 3^b^ | MW646923 | this study |
| *M.* sp. 34 MTP LS 2041^c^ | SAMN15061043 | Brand et al. (2022a) |
| *M. spirale* | KP730565 | Janssen et al. (2015) |
| *M. taurinum* 1^a^ | ON207844 | this study |
| *M. taurinum* 4 | ON207845 | this study |
| *M. taurinum* 5 | ON207846 | this study |
| *M. tuba* | KP730586 | Janssen et al. (2015) |
| *M. zhaoqingense* 1 | KY006974 | Lin et al. (2017b) |
| *M. zhaoqingense* 2 | KY006975 | Lin et al. (2017b) |
| *M. zhaoqingense* 3 | KY006976 | Lin et al. (2017b) |
| *M. zhujiangense* 1 | ON207847 | this study |
| *M. zhujiangense* 2 | ON207848 | this study |
| *M. zhujiangense* 3 | ON207849 | this study |
| *Psammomacrostomum* sp. 5 | KP730585 | Janssen et al. (2015) |

^a^ The same individuals used in Zhang et al. (2021). ^b^ The same individuals used in Xin et al. (2019). ^c^ Sequences obtained from transcriptomes. The accession number is transcriptome accession number.
