# Supplementary figures and images for: ﻿Three new species of Macrostomum (Platyhelminthes, Macrostomorpha) from China and Australia, with notes on taxonomy and phylogenetics
Source: Zookeys. 2022 May 3;1099:1–28. doi: 10.3897/zookeys.1099.72964 (PMC9848920; doi:10.3897/zookeys.1099.72964)

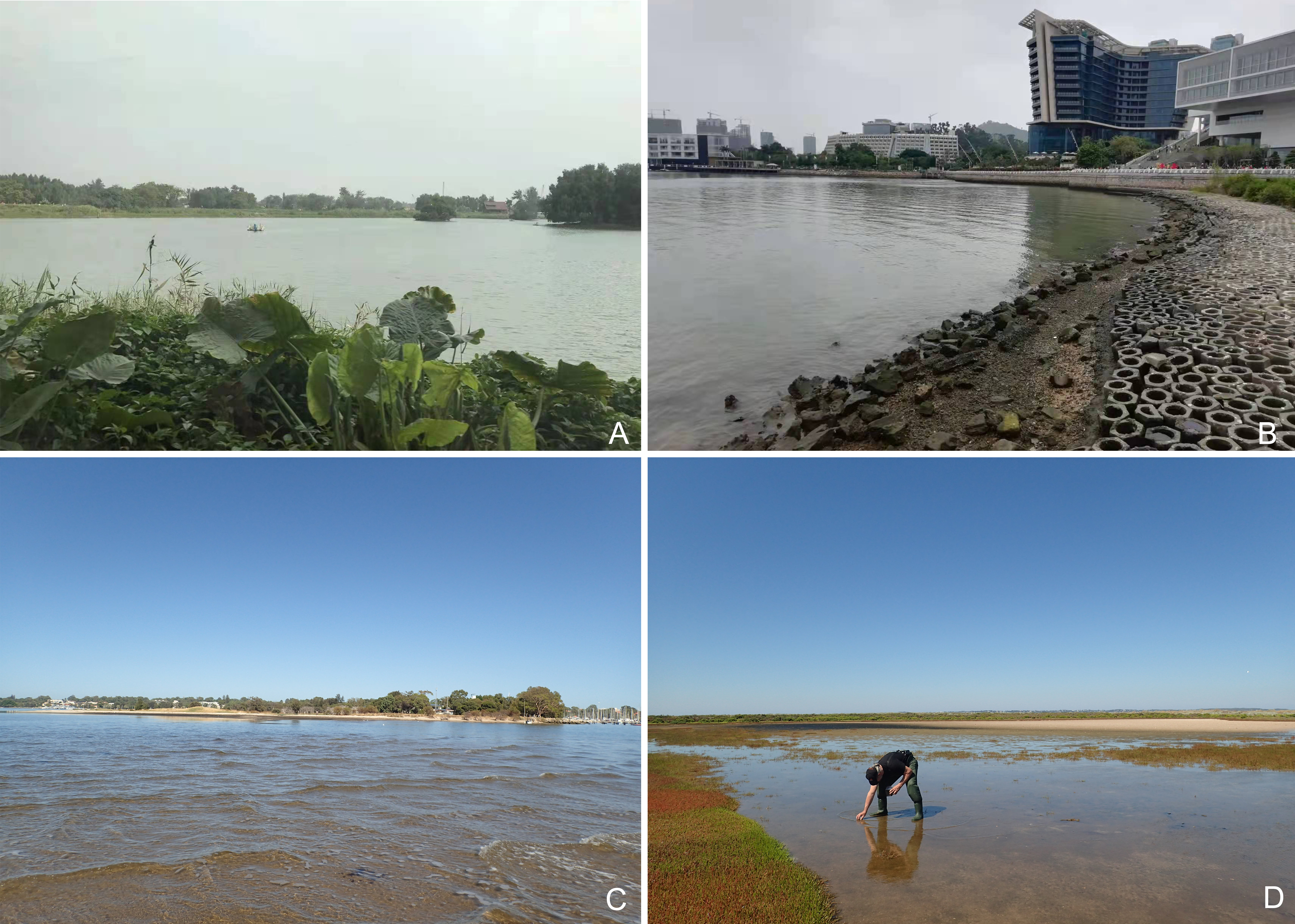

Supplement: Supplementary material 4 — Figure S1 [file zookeys-1099-001_article-72964__-s004.jpg]

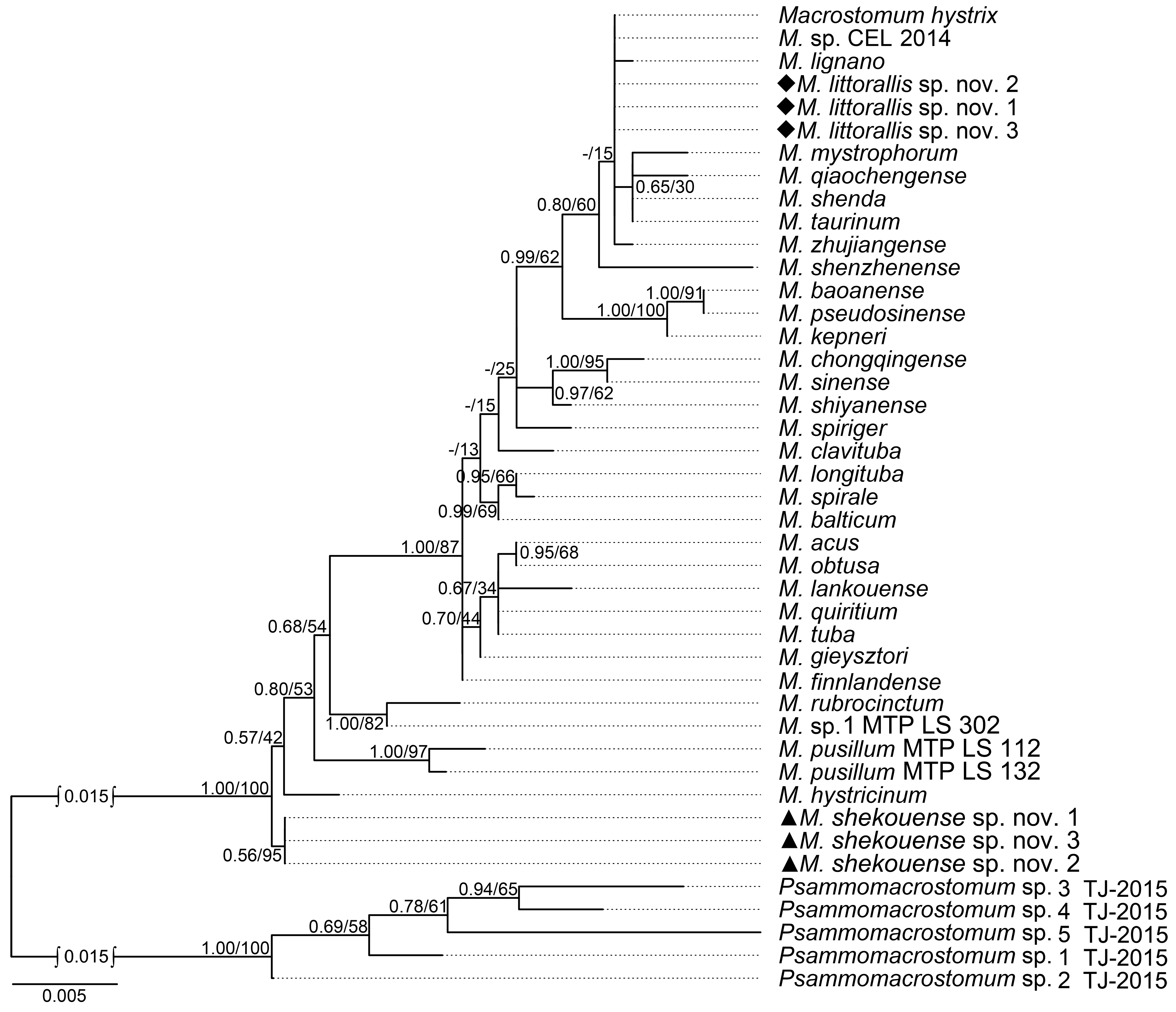

Supplement: Supplementary material 5 — Figure S2 [file zookeys-1099-001_article-72964__-s005.jpg]

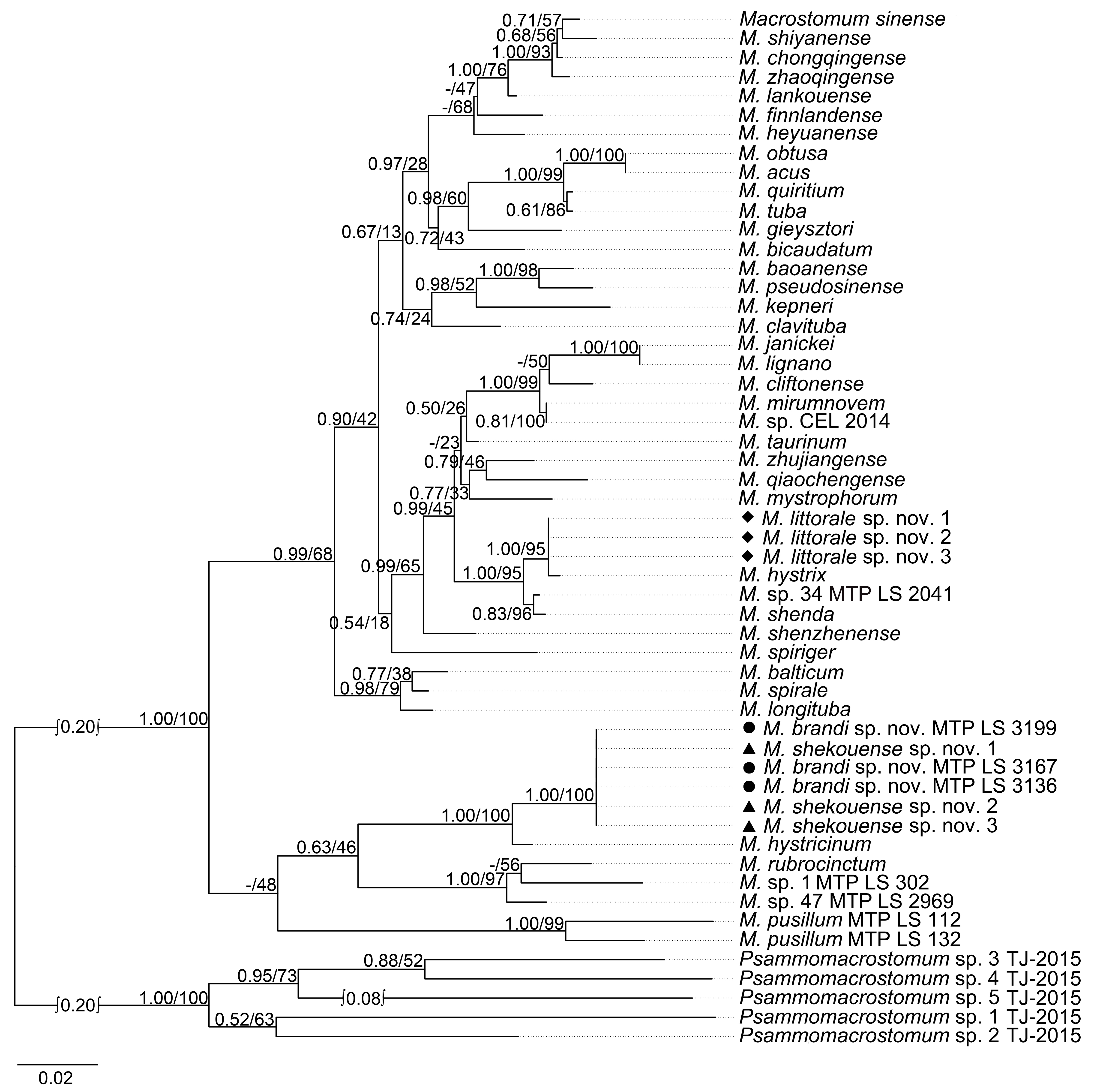

Supplement: Supplementary material 6 — Figure S3 [file zookeys-1099-001_article-72964__-s006.jpg]

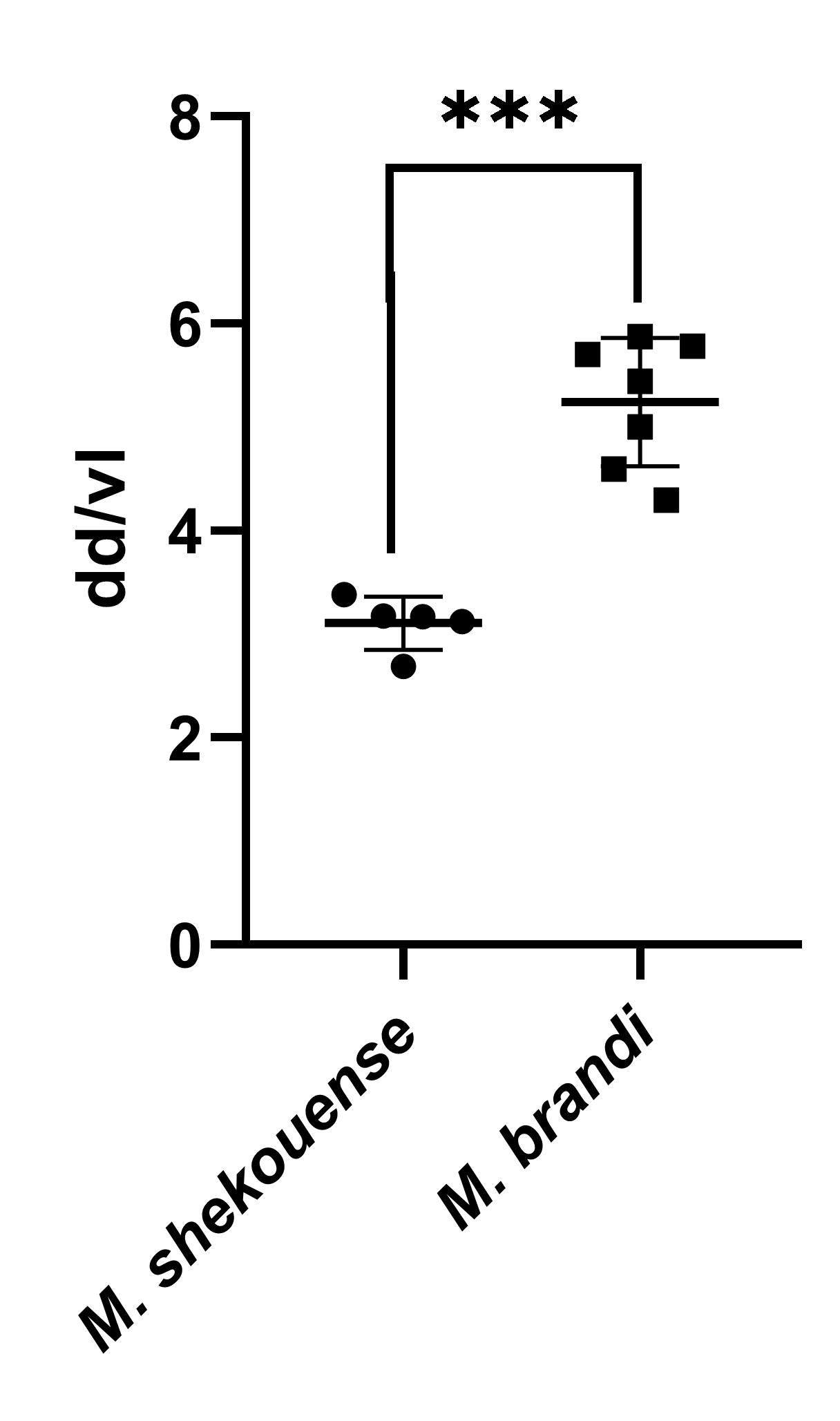

Supplement: Supplementary material 7 — Figure S4 [file zookeys-1099-001_article-72964__-s007.jpg]
